# Impact of dietary *myo*-inositol supplements on immune cell distribution and cytokine expression in laying hens of two different strains

**DOI:** 10.1016/j.psj.2026.107575

**Published:** 2026-08-05

**Authors:** Nadine Wallauch, Sonja Schmucker, Tanja Hofmann, Vera Sommerfeld, Martin Hasselmann, Korinna Huber, Markus Rodehutscord, Volker Stefanski

**Affiliations:** Institute of Animal Science, University of Hohenheim, 70599 Stuttgart, Germany

**Keywords:** Peak laying hen, Myo-inositol, Immune system, Leukocyte population, Cytokine profile

## Abstract

*Myo*-inositol (**MI**) is involved in the regulation of key immune cell activities, including signaling pathways and cellular processes. Although immune cell distribution and cytokine expression are key determinants of immune competence in laying hens, the potential role of MI in regulating these parameters is largely unexplored. Thus, this study investigated the effects of dietary MI on the immune system of laying hens. At the age of 26 wk, corresponding to the peak laying period, 40 Lohmann Brown-Classic (**LB**) and 40 Lohmann LSL-Classic (**LSL**) hens received a maize-soybean meal-based diet either without (**MI0**) or with 1 (**MI1**), 2 (**MI2**) or 3 (**MI3**) g supplemented MI/kg feed for 4 wk, resulting in a 2 × 4 factorial design. We measured numbers of various leukocytes in blood, spleen and cecal tonsils, lymphocyte functionality and gene expression of pro- and anti-inflammatory cytokines. Dietary MI supplementation resulted in cell type-, strain- and concentration-specific effects, including increased circulating B cells in LSL hens fed with MI2, reduced splenic B cells in LB hens with MI1 and MI2 and lower CD4^+^CD25^high^ cells in the cecal tonsils of both strains with MI2. Strain-specific modulation of foxp3 and IL-12 gene expression was also observed. However, neither diet × strain interaction nor dietary MI revealed a clear dose-response relationship. On the contrary, immunological differences between the two strains, irrespective of MI supplementation, were highly prominent and are consistent with previous studies, with a generally higher innate immune response in LB hens, and a more specific and cellular immune response in LSL hens. Overall, our findings indicate that dietary MI supplementation does not broadly alter immune responses in laying hens but can modulate specific immune parameters in a strain-dependent manner. Despite the limited impact, dietary MI may still represent a potential approach to support more balanced immune responses. The results further emphasize immunological, physiological and metabolic variations between the two strains and the importance of genetic background when evaluating nutritional strategies for immune modulation.

## Introduction

Feed additives, such as minerals, amino acids or probiotics, are important management tools in poultry production and have long been used to enhance productive traits, modulate immunocompetence and improve animal health and welfare in chickens. Thereby, as the most prevalent form of inositol (cyclohexane-1,2,3,4,5,6-hexol), *myo*-inositol (**MI**) plays a crucial role in poultry metabolism. It was found to be involved in several metabolic pathways, the central nervous system, cell nuclear processes, cell signaling pathways and membrane functions (Huber, 2016; Gonzalez-Uarquin et al., 2020; Su et al., 2023). Moreover, in mammalian species, MI is directly associated with various effector functions in immune cells through its role in second messenger signaling pathways (Feske, 2007; Suzuki et al., 2010). MI serves as a precursor of phosphatidylinositol (**PI**), phosphoinositides (**PIP**), and inositol phosphates (**InsP_x_**), which are involved in membrane turnover and intracellular signaling pathways (López-Gambero et al., 2020; Gonzalez-Uarquin et al., 2021). In this context, MI as well as lower InsP_x_-derived second messenger cascades, such as the PIP_3_ signal cascade are of particular importance for immune signaling pathways (Huber, 2016). MI availability is regulated by dietary intake and endogenous synthesis. It can be obtained from dietary sources, such as phytate degradation in the digestive tract or free MI supplementation or can be generated by endogenous de novo synthesis from glucose metabolism and dephosphorylation of inositol-containing membrane phospholipids (Croze and Soulage, 2013; Gonzalez-Uarquin et al., 2020). Catabolism of MI occurs mainly in the kidney, contributing to the regulation of MI homeostasis (Croze and Soulage, 2013).

The immune-modulating capacity of MI and its influence on inflammation processes have been described in humans and model organisms but have not been investigated in laying hens so far. Bartnicki et al. (1995) and Wood et al. (2011) showed that, in mammals, MI can be internalized by different immune cells. Given its function as a structural basis of important second messengers, MI participates in the development of T cells, B cells and natural killer (**NK**) cells, as well as in tolerance induction and in the function of neutrophils, mast cells and NK cells, as reviewed in several studies (Feske, 2007; Miller et al., 2008; Sauer and Cooke, 2010). Moreover, MI has been suggested to influence inflammatory processes and immune responses. In a murine lung cancer model, dietary MI supplementation was associated with reduced IL-6 levels and altered macrophage-related immune functions (Unver et al., 2018). Based on the proposed role of IL-6 in inflammatory responses, MI has further been suggested as a potential modulator of IL-6-associated inflammatory pathways and cytokine cascades (Bizzarri et al., 2020). In addition, observations in patients with autoimmune thyroiditis suggest immunomodulatory effects of MI in combination with seleno-methionine, including a potential influence on type 1 T helper cell-associated immune responses (Ferrari et al., 2017).

Studies in poultry investigating MI supplementation are often limited to broiler chickens and their performance traits. In broilers, dietary supplementation of free MI increased plasma MI concentrations, following MI supplementation with 3.5 g/kg feed (Sommerfeld et al., 2018) or 30 g/kg feed (Pirgozliev et al., 2019) Furthermore, higher concentrations of plasma serotonin and dopamine, as well as increased metabolic performance were observed when 3.5 g MI/kg feed was supplemented (Gonzalez-Uarquin et al., 2020). Although studies in laying hens are rare, Herwig et al. (2019, 2021) observed comparable effects, including a tendency towards increased plasma MI concentrations and a decrease in fear behavior after MI supplementation, indicating a similar potential impact on laying hens. Recent studies in laying hens also suggest that genetic background plays a role, as distinct variations were observed between the strains Lohmann Brown-Classic (**LB**) and Lohmann LSL-Classic (**LSL**) in terms of metabolic pathways and intestinal MI concentrations (Gonzalez-Uarquin et al., 2021; Sommerfeld et al., 2025). Likewise, immunological differences exist between these strains, with a stronger innate and humoral immunity in LB hens and a more pronounced specific and cellular immune response in LSL hens (Hofmann et al., 2021; Schmucker et al., 2021).

Recently, Hofmann et al. (2026) investigated the immunomodulatory potential of MI in broiler and showed that in ovo administration of 24 µmol/mL MI resulted in lower plasma IgM concentrations in 35-day old broiler, suggesting an influence of MI on early B cell activation and primary antibody responses (Hofmann et al., 2026). However, effects of MI supplementation on immune functions and inflammatory processes in laying hens are still unknown. This study therefore investigated the effects of different dietary MI concentrations on the systemic and gut-associated immune parameters and on the inflammatory profile in laying hens. As the genetic background has a strong impact on immune pathways and MI metabolism, the two high-performance laying hen strains LB and LSL were included.

## Materials and methods

The present study was part of the interdisciplinary Research Unit P-FOWL: “Inositol phosphates and *myo*-inositol in the domestic fowl: Exploring the interface of genetics, physiology, microbiome, and nutrition” (https://p-fowl.uni-hohenheim.de/). The study was approved by the animal ethics committee (Regional Council Tübingen, Germany (Project no. HOH67-21TE) and conducted in accordance with German animal welfare legislation. The animal trial was carried out at the Agricultural Experiment Station of the University of Hohenheim, Germany.

The experimental design has been described in detail by Sommerfeld et al. (2025), including performance data, precise feed composition and physiological parameters related to feeding. In brief, the 2 × 4 factorial design included the factors strain (**LB** and **LSL**) and 4 different concentrations of dietary MI supplementation. MI was supplemented at concentrations of 0 g/kg MI (**MI0**), 1 g/kg MI (**MI1**), 2 g/kg MI (**MI2**) or 3 g/kg MI (**MI3**). Supplementation levels were chosen in order to simulate approximately half or complete release of MI contained in the feed as InsP_6_ (1 and 2 g MI/kg) or as the therapeutic dose level applied in humans (3 g MI/kg).

Experimental diets were based on corn and soybean meal and were calculated to contain 2.2 g non-phytate P, based on recent suggestions (Rodehutscord et al., 2023), and adequate levels of all other nutrients according to the recommendations of the German Society of Nutrition Physiology. A detailed composition of the experimental diets according to Sommerfeld et al. (2025) can be found in Supplementary Table S1. At the age of 26 wk, hens were placed into metabolic units and received one of the 4 experimental diets for a total of 4 wk, a period considered sufficient to evaluate short-term immunological responses and to provide initial evidence of immune modulatory effects of dietary MI in laying hens. The diets were evenly distributed across the strains, resulting in an experimental setup with 10 replicates per strain and diet (n = 80), in a randomized complete block design. Feed and water were provided ad libitum.

### Blood and tissue sampling

For the immunological analyses we collected blood, spleen as the major secondary lymphatic organ, the cecal tonsils (**CT**) as important lymphatic tissue in the intestine, bile and parts of the oviduct. In order to exclude any influence of the slaughter process on blood immune cell numbers, blood samples were taken by puncturing the vena ulnaris 1 wk before slaughter at the age of 29 wk, on two consecutive days. Blood was taken on average of 1 min 37 s and at least within 4 min after the hens' removal from the pen, thereby minimizing the effects of an acute stress response to capture and handling on immune cell distribution in the blood. Blood was collected in 5 mg/ml EDTA (Sigma Aldrich, St. Louis, MO), fixed using TransFix® reagent (#TFB-01-1, Cytomark Ltd., Buckingham, UK) according to the manufacturer's instructions, and stored at room temperature until further processing for flow cytometric analysis.

At the age of 30 wk, tissue and bile samplings were conducted on 4 consecutive days, with treatments evenly distributed over the days. Hens were individually stunned with a gas mixture of 35 % CO_2_, 35 % N_2_ and 30 % O_2_ and killed by bleeding. Trunk blood was collected in tubes containing EDTA, centrifuged (10 min, 2500 × *g*) and the plasma was stored at −20°C until further processing for immunoglobulin (**Ig**) analysis. Immediately after slaughter, the spleen and one randomly chosen CT was collected and stored in PBS (Roth, Karlsruhe, Germany) containing 1 % Fetal Bovine Serum (**FBS**) and 50 µg/ml gentamycin (both Sigma Aldrich, St. Louis, MO) on ice for transport. Bile was collected by puncturing the gallbladder, stored on ice for transport and frozen at −20°C until determination of Ig concentrations. The second CT and parts of the oviduct were taken and directly shock frozen with liquid nitrogen. Frozen samples were transported on dry ice and stored at −80°C until further analysis by quantitative reverse transcription PCR (**qRT-PCR**).

### Sample preparation

Spleen and CT were processed according to Hofmann and Schmucker (2021) to separate immune cells from the lymphoid tissues for flow cytometric analysis and functional assays. The weight of spleen and one randomly selected CT, was determined. Intraepithelial lymphocytes (**IEL**) of the CT were isolated from the mucosa by shaking the CT tissue twice in Hanks' Balanced Salt Solution (without Mg^2+^ and Ca^2+^), supplemented with 5 mM EDTA, 5 % FBS and 1 mM dithiothreitol (#RO862, Thermo Fisher Scientific, Waltham, MA) for 20 min under slow rotation. The suspension was filtered with a 40 µm MACS SmartStrainer (Miltenyi Biotec, Bergisch Gladbach, Germany) and the IEL containing filtrate was washed in two steps. The single cell suspension was centrifuged (10 min at 300 × *g* at 4°C), and the cell pellet was resuspended in PBS containing 1 % FBS. The final volume of the IEL suspension was determined and stored on ice until further processing for flow cytometric analysis. The spleen was cut in half under aseptic conditions. One half was directly frozen in liquid nitrogen and stored at −80°C until further analysis by qRT-PCR. The other half was dissociated using a gentleMACS Dissociator (Miltenyi Biotec, Bergisch Gladbach, Germany) and the obtained suspension was passed through a 40 µm MACS SmartStrainer, and subsequently through a 20 µm MACS SmartStrainer (both Miltenyi Biotec, Bergisch Gladbach, Germany) to clear cells from connective tissue. After a centrifugation step (10 min at 300 × *g* at 4°C), the cell pellet was resuspended in PBS with 1 % FBS, and the final volume of the spleen lymphocyte suspension was determined. A part of each sample (1 mL per hen) was separated and stored on ice until further processing for flow cytometric analysis. Remaining splenocytes were further processed by gradient centrifugation as described by Hofmann et al. (2021) to separate mononuclear cells for functional analysis. The cell suspension was loaded onto a gradient (1.077 g/mL Biocoll separation solution, Biochrom, Berlin, Germany) and centrifuged (12 min at 600 × *g* at 20°C). Mononuclear cells were washed in PBS + 1 % FBS and resuspended in RPMI 1640 + 10 % FBS. Isolated mononuclear leukocytes were immediately processed for lymphocyte proliferation assay.

### Flow cytometric analysis

Flow cytometric analysis was performed on a BD FACSCanto^TM^ II (BD Bioscience, Heidelberg, Germany) equipped with a 488 nm blue laser, 630 nm red laser and 405 nm violet laser using BD FACSDiva^TM^ Software II (DB Bioscience). Leukocyte subsets in blood, spleen and CT were differentiated and enumerated using a no-lyse-no-wash method as already described in Seliger et al. (2012) and Hofmann and Schmucker (2021) with some adaptations according to Hofmann et al. (2025) and Wallauch et al. (2026). The following chicken-specific antibody-fluorochrome conjugates were used: Anti-CD45-APC (#8270-11, clone LT40), anti-monocyte/macrophage-PE (#8420-09, clone Kul01), anti-CD4-PacBlu-PE or -PE.Cy7 (#8210-26; #8210-09;#8210-17, clone CT-4), anti-CD3-PE (#8200-09, clone CT-3), anti-CD8a-FITC (#8220-02, clone CT-8), anti-Bu-1-FITC (#8395-02, clone AV20) (all SouthernBiotech, Birmingham, AL), anti-CD41/61-PE (#MCA2240PE, clone 11C3; BioRad, Hercules, CA), anti-TCRγδ-PerCP (#NBP1-28275PCP, clone TCR1, Novus Biologicals, Centennial, CO) and anti-CD25 (#MCA5925GA, clone AV142 Biorad, Hercules, CA), customer-coupled to Alexa647 using an Alexa Flour® 647 Antibody Labeling Kit (#A20186 Invitrogen, ThermoFisher Scientific, Waltham, MA, USA), all used according to the manufacturers' instructions. A mouse IgM isotype control conjugated to APC (#0101-11, clone 11E10; Southern Biotech) was used to exclude non-specific binding of anti-CD45-APC and to ensure the accurate enumeration of total leukocyte count. The antibody panel of Hofmann and Schmucker (2021) for labelling single cell suspensions of splenocytes and IEL from CT was extended with the following antibodies: Anti-CD3-PE for differentiation between T cells and non-T cells for the subsequent differentiation between cytotoxic T cells (**CTL**) and natural killer (**NK)** cells, as well as anti-CD25-Alexa647 (s. above) for differentiation of potential regulatory T cells (**Tregs**), as described by Hofmann et al. (2025) and Wallauch et al. (2026). Specific immune cell types were classified by the combination of surface marker expressions as follows: Total leukocytes (CD45^+^), thrombocytes (CD45^dim^/CD41/61^+^) (blood and spleen only), monocytes (CD45^+^/Kul01^+^) (blood and spleen only), NK cells (CD45^+^/CD3^-^/CD8a^+^) (spleen and CT only), B cells (CD45^+^/Kul01^-^/Bu-1^+^), CD4^+^ T cells (CD45^+^/CD3^+^/ CD4^+^/TCRγδ^-^/CD8a^+^ or CD8a^-^), γδ T cells (CD45^+^/CD3^+^/CD4^-^/TCRγδ^+^/CD8a^+^ or CD8a^-^), CTL (CD45^+^/CD3^+^/CD4^-^/TCRγδ^-^/CD8α^+^), CD4^+^/CD25^-^ cells, CD4^+^/CD25^dim^ cells, and potential Tregs (CD4^+^/CD25^high^). Heterophils in the blood were identified based on their FSC/SSC characteristics.

In each case, 50 µL pre-diluted blood (1:50) or 50 µl of spleen and CT tissue sample were stained with the respective antibody mix in a total volume of 70 µL for 45 min at room temperature according to Hofmann and Schmucker (2021). SYTOX Blue Dead Cell Stain (# S34857, Invitrogen, Thermo Fisher Scientific, Waltham, MA, USA) was used to exclude dead cells in cell suspensions of splenocytes and IEL of CT. Cells were suspended in 1000 µL (splenocytes) or 400 µL (whole blood and IEL of CT) PBS supplemented with 2 % BSA, 0.1 % NaN_3_ and 5 mg/mL EDTA and stored at 6°C until analysis. A minimum of 10,000 CD45^+^ cell events of whole blood and 30,000 CD45^+^ cell events of tissue sample were analyzed based on the respective combination of surface marker expressions after exclusion of cell doublets. BD Trucount™ tubes (#340334; BD Biosciences, Heidelberg, Germany) were used according to the manufacturer's instructions to determine the absolute number of leukocytes per µL blood or tissue. Total leukocyte count was combined with determined cell frequencies to calculate the absolute cell counts of each leukocyte subset.

### Splenic lymphocyte proliferation assay

An in vitro mitogen-induced lymphocyte transformation assay was used to quantify splenocyte proliferation capacity, as described in detail by Hofmann et al. (2021). A final concentration of 1.5 × 10^5^ mononuclear cells per animal were seeded per well of a 96-well round-bottom cell culture plate (Neolab, Heidelberg, Germany) and were stimulated for 44 h with either 10 µg/mL concanavalin A (ConA), 10 µg/mL poke weed mitogen (PWM) (both Sigma Aldrich, St. Louis, MO), or left unstimulated as a negative control at 41°C and 5 % CO_2_. Each treatment was performed in triplicate. Subsequently, 0.25 µCi ^3^H-thymidine (PerkinElmer, Rodgau, Germany) was added to each well and cells were further incubated for 24 h. Cells were harvested on glass fiber filters (Skatron, Lier, Norway) and the amount of incorporated radioactivity was evaluated using a liquid scintillation analyzer (PerkinElmer, Rodgau, Germany). The mean of cpm was calculated for each triplicate, and delta cpm for ConA- and PWM-stimulated splenocytes was determined (= mean cpm of stimulated cells - mean cpm of non-stimulated cells). The inter-assay coefficient of variation (**CV**) for delta cpm of ConA was 23.5 % and 18.4 % for delta cpm of PWM.

### Enzyme-linked immunosorbent assay

Concentrations of IgM, IgY and IgA antibodies in trunk blood plasma and of IgA in bile were detected by ELISA as described by Hofmann et al. (2021). As primary antibodies, goat anti-chicken IgA Fc (#A30-103-A), goat anti-chicken IgM Fc (#A30-102-A) and goat anti-chicken IgY Fc (#A30-104-A) (all Bethyl Laboratories, Montgomery, TX) were used. A final concentration of either 200 ng/well for IgA and IgM, or 100 ng/well for IgY, was coated on 96-well flat-bottom microtiter plates (Thermo Fisher Scientific, Waltham, MA). Plasma sample dilutions (IgA: 1:2,000, IgM: 1:20,000, IgY: 1:400,000) and bile samples (IgA: 1:1,000,000) were applied in triplicates, respectively. Captured antibodies were detected using either 2 ng/well of horseradish peroxidase-labelled goat anti-chicken IgA Fc (#A30-103-P), or 1 ng/well goat anti-chicken IgM Fc (#A30-102-P), or goat anti-chicken IgY Fc (#A30-104-P) (all Bethyl Laboratories, Montgomery, TX) as detection antibodies. After 1 h of incubation, tetramethylbenzidine (AppliChem, Darmstadt, Germany) was added and color formation was stopped after 20 min with 2 M H_2_SO_4_ (Roth, Karlsruhe, Germany). All samples were quantified by reference to a calibration curve set up with pooled plasma controls based on known concentrations of chicken IgA, IgM or IgY, as previously determined using chicken IgG ELISA Kit (#E33-104), Chicken IgM ELISA Kit (#E33-102) and Chicken IgA ELISA Kit (#E33-103) (all Bethyl Laboratories, Montgomery, TX, USA). Absorbance was measured at 450 nm. Antibody concentrations were calculated relative to the absorbance of the calibration curve. The intra-assay CV was 3.1 % for plasma IgA, 3.8 % for plasma IgM, 6.7 % for plasma IgY and 4.3 % for bile IgA. The inter-assay CV was 7.2 % for plasma IgA, 9.4 % for plasma IgM, 7.7 % for plasma IgY and 2.4 % for bile IgA.

### Gene expression analysis by quantitative reverse transcription PCR

Gene expression analysis in spleen, CT and the oviduct was performed using a high-throughput gene expression method as already described by Wallauch et al. (2026). In brief, RNA of 100 mg tissue was extracted using Trizol Reagent (#15596018, Invitrogen, Thermo Fisher scientific Inc., Waltham, MA) according to the manufacturer’s protocol. RNA concentration and quality were measured using a NanoDrop2000 Spectrophotometer (Thermo Fisher scientific Inc., Waltham, MA), provided in Supplementary Table S2 and S3. Moreover, RNA integrity of a random but representative subset (including all tissues and treatments) was checked via gel-electrophoresis (Aranda et al., 2012) and on Qubit 4, using the Qubit RNA IQ Assay Kit (#Q33221) (both Thermo Fisher Scientific, Waltham, MA), provided in Supplementary Table S4. Samples were stored at −80°C until further processing.

For qRT-PCR primers of 17 genes were selected for qRT-PCR. This included genes of ten cytokines (IL-1β**,** IL-2, IL-4, IL-6, IL-10, IL-12, IL-13, IL-17, tumor necrosis factor alpha (**TNF-α**), interferon-gamma (**IFN-γ**)), the master transcription factor of Tregs (Forkhead Box P3 (**Foxp3**)), and three genes related to oxidative stress reactions (inducible nitric oxide synthase (**iNos**) and superoxide dismutase (**SOD**) 1 and 2). In addition, three potential nuclear-encoded reference genes (Beta-actin (**ACTB**), peptidylprolyl isomerase A (**PPIA**) and Glyceraldehyde-3-phosphate dehydrogenase (**GAPDH**)), were included. A list of the final genes and related primers used in this work can be found in Supplementary Table S5. In advance, primer specificity, limits of detection, linear dynamic detection range, variation at detection limit, PCR efficiency and melting curves of each primer product were determined. The final qRT-PCR was performed on Biomark HD (Standard Biotools, South San Francisco, CA 94080, USA). A DNaseI treatment (#EN0525, Thermo Fisher scientific Inc. Waltham, MA) was included starting with a total amount of 2 µg RNA in each reaction. Subsequent cDNA synthesis, pre-amplification and final qPCR runs were performed using Delta Gene Assays protocol with the manufacturer's standard protocol for fast PCR and melting curve (see Supplementary Table S6), on a 96.96 Dynamic Array™ IFC for Gene Expression (Standard Biotools, South San Francisco, CA). Each primer was tested in triplicates. A negative control was included throughout all preparation steps to test for contamination of the primers and reagents.

Data evaluation and quality control was performed using the Standard Biotools Real-Time PCR analysis software (version 1.0.2). The peak ratio threshold was set to 0.8 and the quality threshold to 0.65. Only quantification cycles (**Cq**) from reactions with logarithmic increase of fluorescence and specific melting temperatures were used. In addition to the automatic quality check of the software, the data sets were evaluated by eye. Normfinder was used to evaluate stability of the three potential reference genes for normalization (ACTB, PPIA and GAPDH) under experimental conditions (Andersen et al., 2004). Normalization was tested for strain and diet. Under experimental conditions, a combination of GAPDH and ACTB for spleen and CT, or a combination of GAPDH and PPIA for the oviduct were identified to be the best reference genes for normalization. Means of triplicates were calculated for all samples. For samples with only two or one successful triplicate, the successful runs were used. The Cq values of the genes of interest were normalized to the average Cq values of the reference genes resulting in ΔCq values. ΔCq values were calculated as follows: ΔCq = mean Cq _(gen of interest)_ – mean Cq _(reference genes)_.

### Statistical analysis

For statistical analysis, a linear mixed model was applied, using the software SAS Version 9.4 (SAS Institute Inc., Cary, NC, USA) and the PROC MIXED method. Transformations were used to stabilize variance and to meet the distributional assumption. Individual hens were considered as the experimental unit. The following model was used:

$$Y_{ijkl}=\mu +\alpha_{i}+\beta_{j}+{\left(\alpha \beta \right)}_{ij}+\gamma_{k}+\varphi_{l}+\varepsilon_{ijkl,}$$

where Y_ijkl_ = response variable, µ = overall mean, α_i_ = effect of strain (fixed), β_j_ = effect of MI supplementation (fixed), (αβ)_ij_ = the interaction between strain and MI supplementation (fixed), γ_k_ = block (random), φ_l_ = father/rooster (random) and ε_ijkl_ = residual error. When the main effect of dietary MI supplementation was significant, orthogonal polynomial contrasts were used to assess linear and quadratic dose-response trends. For significant diet × strain interactions, these contrasts were additionally performed separately within each strain.

To consider probable effects of sampling duration on immune parameters, the duration of blood sampling from the vena ulnaris was included as a covariate for statistical analysis. For splenocyte proliferation and antibody ELISA, the model included the respective well plate during analysis as a further random effect. Covariables were checked for significance and removed from the model if they were not significant. Residuals were tested for normal distribution and homogeneous error variance via graphical check of residual plots (Kozak and Piepho, 2018). Statistical significance was declared at P < 0.05. Results are presented as Least Square Means (**LSmeans**) and Standard Error of the Mean (**SEM**) of untransformed or retransformed data. However, LSmeans of ΔCq values of qPCR results were transformed using 2^-ΔCq^ to facilitate interpretation and graphical presentation. Moreover, genes from expression analysis, which were only expressed in a minor proportion of the samples, were analyzed using a generalized linear model with the PROC GLIMMIX method and logit link function to determine differences in their expression frequency. The underlying model structure was based on the linear mixed model of the other evaluations. Binomial distribution was assumed. In order to adequately model the small number of cases, continuous expression data were converted into binary variables (expressed: yes/no). Model quality was assessed using the ratio of Pearson Chi² to the degrees of freedom (Chi²/df).

## Results

The results section focuses on the effects of dietary MI supplementation on immune parameters. Consistent strain differences between LB and LSL hens were also observed (see Supplementary Tables S7–S16). As these differences align with our previous observations (Hofmann et al., 2021; Hofmann and Schmucker, 2021), they are not addressed here unless they interacted with dietary MI supplementation.

### Impact of dietary MI supplementation and genetic strain on the distribution and total cell counts of immune cells in blood and lymphatic tissue

**Fig. 1:**
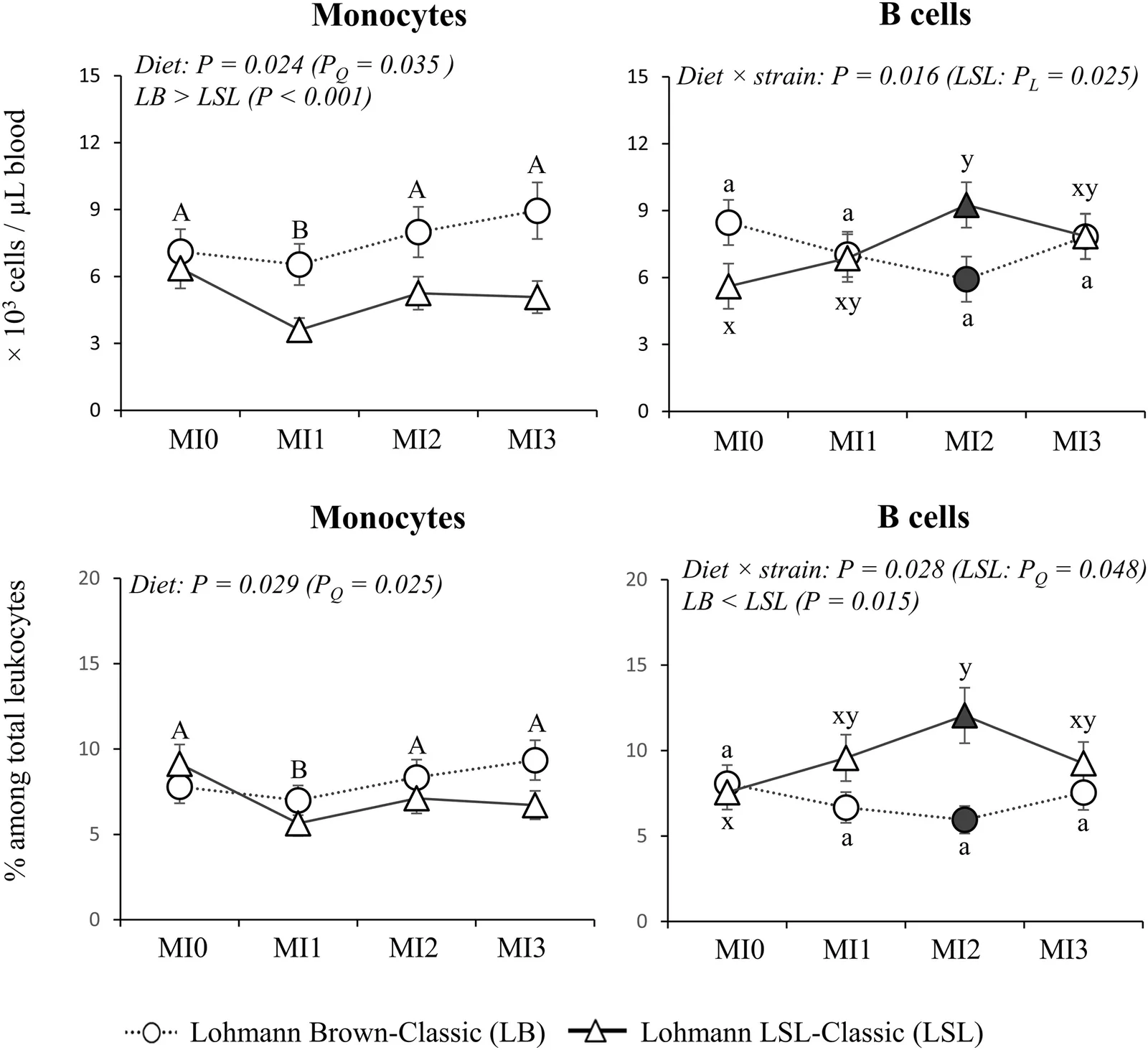
Blood cell populations affected by the impact of dietary MI levels (MI0 = without MI supplementation, MI1 = 1 g supplemented MI/kg, MI2 = 2 g supplemented MI/kg, MI3 = 3 g supplemented MI/kg) and strain (LB = Lohmann Brown-Classic; LSL = Lohmann LSL-Classic). Results of statistical analysis of interactions (diet × strain) and main effects of dietary MI or strain as well as linear (L) or quadratic (Q) polynomial responses are given in each diagram (P < 0.05). P values not shown are ≥ 0.05. Data are presented as LSmeans ± SEM. In case of significance (P < 0.05), interactions are indicated by different lowercase letters for differences between MI concentrations within the same strain (LB: a-c, LSL: x-z), and differences between the strains at particular MI concentrations are indicated by filled-in symbols. Lines are included to better illustrate interactions of diet × strain. Main effects of the diet irrespective of the strain are indicated by different uppercase letters in case of missing interactions. Directions of strain effects are given above. Post hoc comparisons were performed using Fisher LSD test (P < 0.05).

**Fig. 2:**
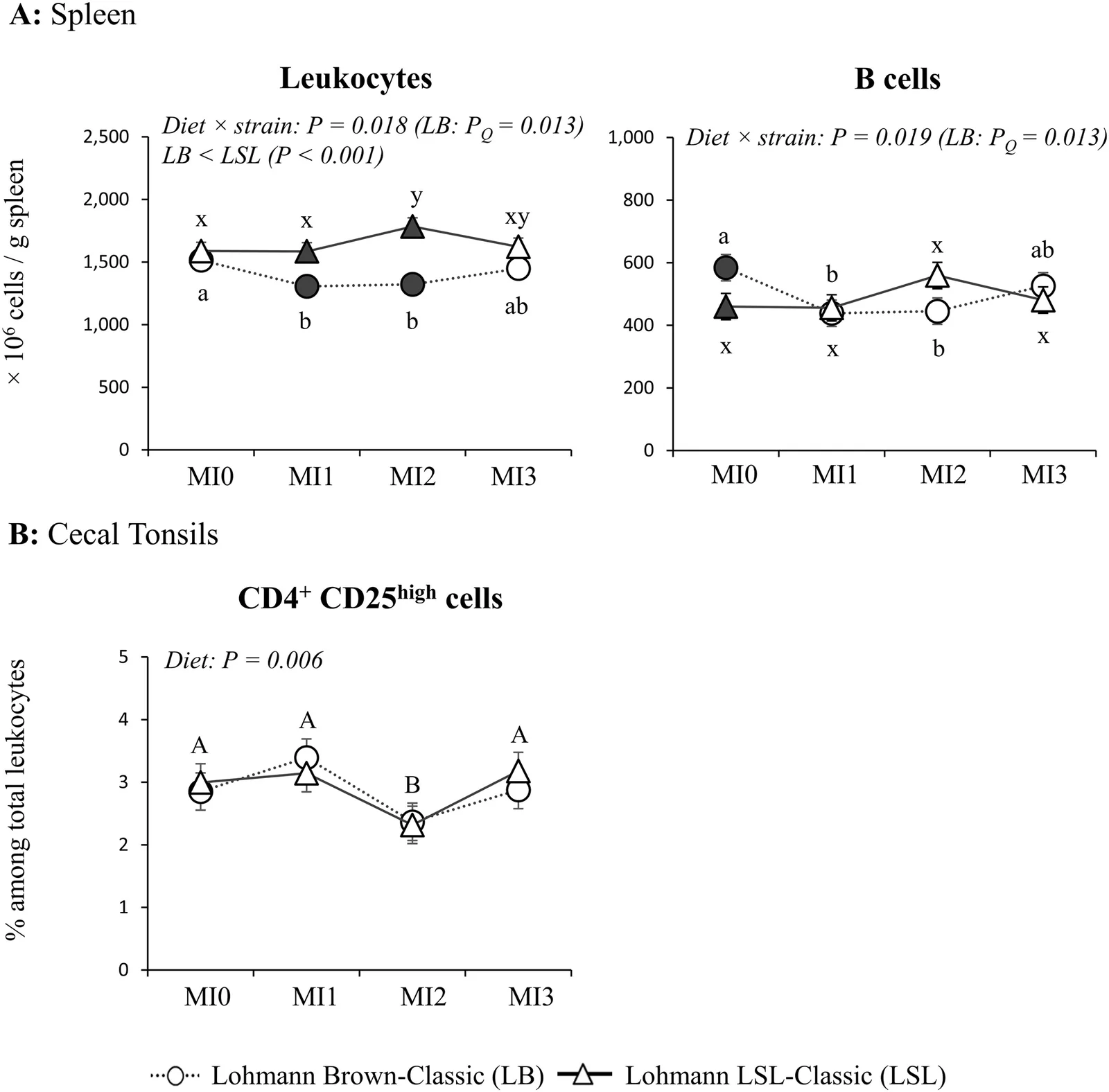
Immune cell populations of spleen (A) and cecal tonsil (B) affected by the impact of dietary MI levels (MI0 = without MI supplementation, MI1 = 1 g supplemented MI/kg, MI2 = 2 g supplemented MI/kg, MI3 = 3 g supplemented MI/kg) and strain (LB = Lohmann Brown-Classic; LSL = Lohmann LSL-Classic). Results of statistical analysis of interactions (diet × strain) and main effects of dietary MI or strain as well as linear (L) or quadratic (Q) polynomial responses are given in each diagram (P < 0.05). P values not shown are ≥ 0.05. Data are presented as LSmeans ± SEM. In case of significance (P < 0.05), interactions are indicated by different lowercase letters for differences between MI concentrations within the same strain (LB: a-c, LSL: x-z), and differences between the strains at particular MI concentrations are indicated by filled-in symbols. Lines are included to better illustrate interactions of diet × strain. Main effects of the diet irrespective of the strain are indicated by different uppercase letters in case of missing interactions. Directions of strain effects are given above. Post hoc comparisons were performed using Fisher LSD test (P < 0.05).

Fig. 1, Fig. 2 show the immune cell populations in blood, spleen and CT, which were affected by dietary MI supplementation. Data on all other cell types are given in the Supplementary Tables S7–S12, including a detailed analysis of strain differences.

***Blood.*** A diet × strain interaction affected total B cell counts per µl blood (*F*(3,53.5) = 3.75, *P* = 0.016) and the relative proportion of B cells among total leukocytes (*F*(3,53.6) = 3.28, *P* = 0.028). Polynomial contrast revealed a linear response of total B cell counts to increasing MI levels in LSL hens (*P* = 0.025) and a quadratic response for the relative proportion of B cells in LSL hens (*P* = 0.048). In LSL hens fed the MI2 diet, B cell counts were higher than in LSL hens fed the MI0 diet (*t*(53.2) = −2.79, *P* = 0.007) and in LB hens fed the MI2 diet (*t*(65.5) = −2.32, *P* = 0.024). Similar effects were seen in the relative proportion of B cells. The main effect of dietary MI affected the numbers of monocytes (*F*(3,62) = 3.36, *P* = 0.024), with a quadratic response to increased MI levels (*P* = 0.035). Lower cell counts were observed in hens fed the MI1 diet compared to all other diets (MI0 (*t*(62.1) = 2.70, *P* = 0.009); MI2: (*t*(62.1) = −2.38, *P* = 0.02); MI3: (*t*(62.1) = −2.71, *P* = 0.009). No difference was found between MI0, MI2 and MI3. Moreover, a similar pattern of effects was detected on the relative proportion of monocytes among total leukocytes (*F*(3,46.7) = 3.27, *P* = 0.029), which also showed a quadratic response to increasing MI levels (*P* = 0.025) (Fig. 1).

***Spleen.*** A diet × strain interaction affected the numbers of leukocytes (*F*(3,54) = 3.67, *P* = 0.018) and B cells (*F*(3,49.4) = 3.63, *P* = 0.019) per g spleen. Polynomial contrast revealed a quadratic response to increasing MI levels in LB hens for both leukocytes (*P* = 0.013) and B cells (*P* = 0.013). Higher leukocyte counts were found in LB hens fed the MI0 diet compared to LB hens fed the MI1 (*t*(54) = 2.4, *P* = 0.02) and MI2 diet (*t*(54) = 2.23, *P* = 0.03). In contrast, in LSL hens the MI2 diet led to higher leukocyte counts compared to MI0 (*t*(54) = −2.25, *P* = 0.028) and MI1 (*t*(54) = −2.28, *P* = 0.027). Moreover, LSL hens showed higher leukocyte counts when fed the MI1 diet (*t*(63.2) = −2.84, *P* = 0.006) or MI2 diet (*t*(63.2) = −4.71, *P* < 0.001) compared to LB hens fed the same diet. B cell counts per g spleen were higher in LB hens fed the MI0 diet compared to LSL hens fed the same diet (*t*(56.2) = 2.12, *P* = 0.039) and LB hens fed the MI1 (*t*(49.4) = 2.75, *P* = 0.008) or MI2 diet (*t*(49.4) = 2.63, *P* = 0.011) (Fig. 2A). The main effect of dietary MI had no impact on cell counts per g spleen and the relative proportion of immune cells (Supplementary Tables S9 and S10).

***Cecal Tonsils.*** Neither the diet × strain interaction nor the main effect of dietary MI affected total cell counts per g of CT (Supplementary Table S8). However, the relative proportion of CD4^+^CD25^high^ T cells among total leukocytes (*F*(3,54) = 4.60, *P* = 0.006) was affected by MI supplementation. The frequency of CD4^+^CD25^high^ T cells was lower when fed the MI2 diet compared to MI0 (*t*(54) = 2.24, *P* = 0.029), MI1 (*t*(54) = 3.57, *P* < 0.001) and MI3 (*t*(54) = −2.64, *P* = 0.011) (Fig. 2B).

### Impact of dietary MI supplementation and genetic strain on immune cell functionality

Neither the diet × strain interaction, nor the main effect of dietary MI affected splenocyte proliferation capacity or antibody concentrations in plasma and bile (Supplementary Table S13).

### Impact of dietary MI supplementation and genetic strain on gene expression related to immunological pathways

**Fig. 3:**
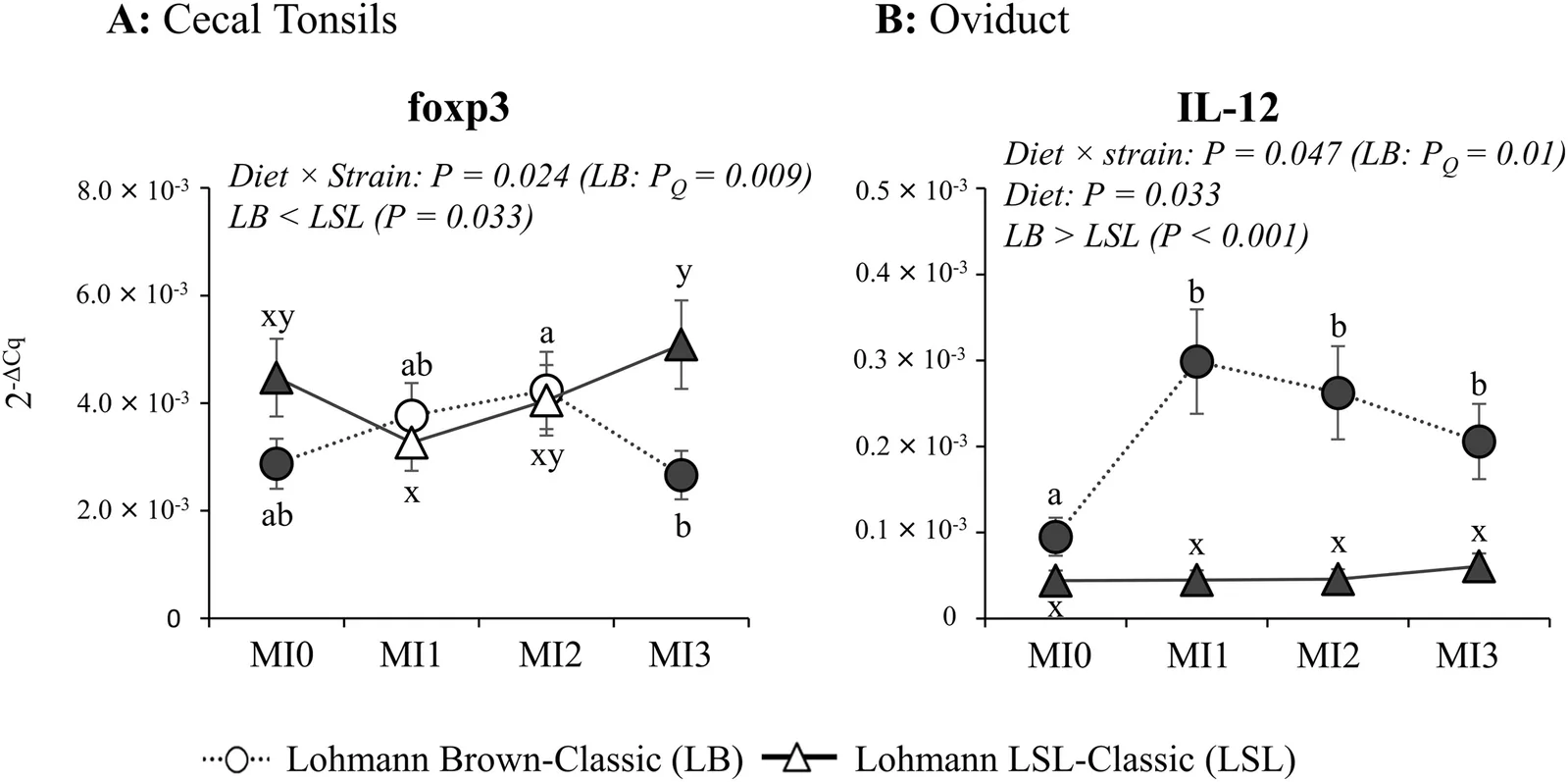
Gene expressions of cecal tonsil (A) and oviduct (B) affected by the impact of dietary MI levels (MI0 = without MI supplementation, MI1 = 1 g supplemented MI/kg, MI2 = 2 g supplemented MI/kg, MI3 = 3 g supplemented MI/kg) and strain (LB = Lohmann Brown-Classic; LSL = Lohmann LSL-Classic). Results of statistical analysis of interactions (diet × strain) and main effects of dietary MI or strain as well as linear (L) or quadratic (Q) polynomial responses are given in each diagram (P < 0.05). P values not shown are ≥ 0.05. Data are presented as 2^-ΔCq^-transformed LSmeans ± SEM of ΔCq values. Lines are included to better illustrate interactions of diet × strain. In case of significance (P < 0.05), interactions are indicated by different lowercase letters for differences between MI concentrations within the same strain (LB: a-c, LSL: x-z), and differences between the strains at particular MI concentrations are indicated by filled-in symbols. Directions of strain effects are given above. Post hoc comparisons were performed using Fisher LSD test (P < 0.05).

Gene expression of pro- and anti-inflammatory cytokines, expression of foxp3 and genes related to oxidative stress reactions were determined in the spleen, CT and the oviduct under different dietary MI concentrations, as a measure of inflammatory conditions. Genes affected by dietary MI supplementation are presented in Fig. 3. Data of all other genes are given in Supplementary Tables S14–S16, including a detailed analysis of strain differences.

***Cecal Tonsils.*** A diet × strain interaction affected the expression of foxp3 (*F*(3,61.2) = 3.37, *P* = 0.024) in the CT. A quadratic response to increasing MI supplementation was observed in LB hens (*P* = 0.009). Higher expression levels were found in LSL hens fed the MI0 diet (*t*(61) = 2.17, *P* = 0.034) or MI3 diet (*t*(61.3) = 3.09, *P* = 0.003), compared to LB hens fed the same diet. Moreover, LB hens fed the MI2 diet showed a higher foxp3 expression in the CTs compared to LB hens fed the MI3 diet (*t*(61.7) = −2.15, *P* = 0.035), whereas in LSL hens, the MI3 diet led to higher expressions compared to the MI1 diet (*t*(61) = 2.17, *P* = 0.034) (Fig. 3). The main effect of dietary MI supplementation had no impact on any of the analyzed genes in the CT (Supplementary Table S14).

***Spleen.*** Gene expression was not affected by diet × strain interaction, nor by the main effect of dietary MI supplementation (Supplementary Table S15).

***Oviduct.*** A diet × strain interaction affected IL-12 expression (*F*(3,62.2) = 2.80, *P* = 0.047). A quadratic response to increasing MI supplementation was observed in LB hens (*P* = 0.01). LB hens fed the MI0 diet showed a lower expression of IL-12 compared to LB hens fed the MI1 diet (*t*(62) = 3.79, *P* < 0.001), the MI2 diet (*t*(62) = 3.34, *P* < 0.001) and the MI3 diet (*t*(62) = 2.50, *P* = 0.015). Moreover, LB hens had a higher expression at each MI level compared to LSL hens fed the same diet, respectively (MI0: *t*(62.7) = −2.23, *P* = 0.03; MI1: *t*(62) = −6.04, *P* < 0.001; MI2: *t*(62) = −5.52, *P* < 0.001; MI3: *t*(62) = −3.85, *P* < 0.001). The main effect of dietary MI also affected IL-12 expression (*F*(3,62.2) = 3.10, *P* = 0.033). Hens fed the MI0 diet showed a lower expression compared to the hens fed the MI1 diet (*t*(62.4) = 2.68, *P* = 0.01), the MI2 diet (*t*(62.4) = 2.41, *P* = 0.019) and the MI3 diet (*t*(62.4) = 2.40, *P* = 0.019) (Fig. 3).

## Discussion

So far, the effects of dietary MI supplementation on the immune system and inflammatory profiles in laying hens have received little attention. Here, we demonstrated that the numbers of monocytes in blood, B cells in blood and spleen, as well as the expression of IL-12 in the oviduct, were affected by MI supplementation. Some of these effects were strain-dependent, particularly an increase of blood B cells in LSL hens with MI2, reduced splenic B cells in LB hens with MI1 and MI2, and increased IL-12 expression in the oviduct of LB hens following any MI supplementation at all levels. Although the magnitude of the effect was not pronounced and limited to a few cell types, the findings suggested at least some modulation of macrophages and B cell-driven immune pathways as a result of dietary MI supplementation in laying hens. Moreover, in the gut-associated immune system, dietary MI supplementation affected the relative proportion of CD4^+^CD25^high^ T cells and the gene expression of foxp3, the master transcription factor of regulatory T cells. The changes in the proportion of CD4^+^CD25^high^ T cells and the expression of foxp3 reaffirmed the relevance of the gut-associated immune system as a central interface of nutritional immune modulation and diet-related immunological alterations, as already shown in a previous study (Wallauch et al., 2026). Regulatory T cells in particular, which can be influenced by dietary composition and metabolites of the gut microbiota, seemed to fulfil a crucial function in nutritional modulations (Furusawa et al., 2013; Lee et al., 2018; Tan et al., 2022). It should also be noted that LB hens may benefit more from MI supplementation, as moderate levels of dietary MI increased the expression of foxp3 in LB hens to the same level as in LSL hens. Irrespective of MI supplementation, LSL hens seemed to have a generally higher capacity of immune regulation in the gut-associated immune system, characterized by higher expression levels of foxp3 and IL-10 compared to LB hens. Beyond the strain-specific immune modulations by dietary MI supplementation, the substantial differences between LB and LSL hens confirmed findings of previous studies (Hofmann et al., 2021; Hofmann and Schmucker, 2021; Wallauch et al., 2026). In general, LB hens displayed a more humoral and innate immunological phenotype, whereas LSL hens are characterized by a stronger adaptive and cellular immune response. This could indicate that dietary MI supplementation contributes to a shift towards a strain-dependent improved immune balance by increasing B cell-driven immunity in LSL hens whereas decreasing it in LB hens. Moreover, differences between the strains were suggested by their endogenous potential to synthesize and metabolize MI, with an assumedly higher absorption capacity in LB hens (Sommerfeld et al., 2020, 2025; Gonzalez-Uarquin et al., 2021). This may result in different conversion of MI metabolites, different activation of cellular signaling pathways and thus strain-specific immune modulations.

The findings of the present study did not reveal a clear dose-dependent response regarding the impact of MI on systemic or gut-associated immune parameters. Nevertheless, linear and quadratic response patterns were observed for distinct immune parameters, indicating that the effects of MI supplementation were dependent on individual immune responses and genetic background of the hens rather than following a consistent dose-response relationship. Immune modulation was mainly observed at 1 or 2 g MI/kg diet, whereas the 3 g MI/kg diet produced barely detectable effects. This observation aligns with Sommerfeld et al. (2025), who reported that increasing amounts of dietary MI showed no linear relationship with plasma MI levels, as comparable plasma MI concentrations were found with 2 and 3 g MI/kg diets. Moreover, a similar pattern of effects on metabolite profiles was detected (Sommerfeld et al., 2025; Szentgyörgyi et al., 2025). Although early studies suggested a highly efficient gastrointestinal uptake of MI, based on minimal fecal recovery (Croze and Soulage, 2013), these findings do not necessarily reflect complete absorption, as microbial utilization or metabolic conversion cannot be excluded (Sommerfeld et al., 2025). More recent evidence across species indicates that intestinal absorption is only partial and may be limited or regulated, including in poultry (Sommerfeld et al., 2018, 2025; Herwig et al., 2019, 2021). Despite the highest dose of MI in our study did not induce immunological changes, previous findings also suggest that excessive doses may even lead to adverse effects. A study in mice demonstrated that low and intermediate doses of exogenous MI increased the host´s ability to eliminate bacteria, whereas high doses reduced this ability, suggesting a dose-dependent variation in response (Chen et al., 2015).

The physiological relevance of the immunological effects was not the focus of the present study and warrants future research. However, based on the results, the overall effect on the immune system appears somewhat limited. Despite a few effects on monocytes and B cells, and a potentially positive impact on immune regulation in LB hens, there was no impact of MI supplementation on T cells or on immune cell functionality, such as lymphocyte proliferation or antibody concentrations. Similarly, Hofmann et al. (2026) observed only limited effects of MI administration on immune parameters in broiler chickens, with lower plasma IgM concentration, suggesting an influence of MI on B cell-mediated immune responses, which aligns with the changes in B cell populations observed in the present study. The limited effect of dietary MI supplementation on health and welfare of laying hens is also consistent with the reports of Herwig et al. (2019) and Sommerfeld et al. (2025), neither of which found any beneficial impact on laying performance.

The lack of a more robust effect of dietary MI supplementation on immune parameters in the present study could be related to several factors. First, endogenous MI synthesis in hens fed the control diet might have been already sufficient for optimal immune functions, resulting in no further effects from dietary MI supplementation. In humans, the MI metabolism is driven predominantly by endogenous MI synthesis rather than dietary intake (Croze and Soulage, 2013). Beneficial effects of dietary MI might thus merely occur in the presence of a MI deficiency or metabolic dysfunction. Although dietary MI supplementation was suggested to have a positive impact on the immune system, inflammatory processes and metabolic pathways (Ferrari et al., 2017; Baldassarre et al., 2021; Valle et al., 2025), studies primarily focused on the impact of MI in cell signaling pathways, using in vitro studies or its therapeutic role in human disease patterns that are mainly limited to metabolic disorders, autoimmune disease or cancer (Unver et al., 2018; Chhetri, 2019). Therefore, it is not clear whether MI induces similar beneficial effects in healthy individuals at all. Second, in most mammalian studies, MI administration periods lasted between 2 and 6 months (Lam et al., 2006; Ferrari et al., 2017; Baldassarre et al., 2021) and thus were considerably longer than the period of 4 wk in our present study. Extending the trial period could therefore lead to more pronounced effects and more conclusive results. Third, the few studies conducted in poultry mainly examined broiler chickens and focused on their performance traits, rather than on the immune system. A positive impact of dietary MI supplementation on body weight gain, metabolism and feed intake of broilers was suggested (Zyła et al., 2004; Cowieson et al., 2013; Sommerfeld et al., 2018). However, broilers and layers have been genetically selected for two different production types, resulting in different metabolic properties, duration of life and nutritional requirements (Koenen et al., 2002). Production phases and performance peaks are at different ages and stages of development, which may explain the variability in response to dietary MI supplementation.

In conclusion, the results of the present study indicate a limited impact of dietary MI supplementation on the immune system of laying hens. Effects that could be shown were related to the hens’ genetic background, thereby providing further evidence of immunological, physiological and metabolic variations between the two strains. Our results did not generally indicate immunological improvements or a distinct anti-inflammatory effect of dietary MI supplementation. However, some benefits related to an increased immune regulation capacity in the gut-associated immune system can be assumed, especially for LB hens. Further studies are needed to evaluate the impact of dietary MI supplementation on the immune system of laying hens, especially due to their differences in the genetic background and metabolic pathways. Additional relevant factors, such as the overall metabolic state and presence of dysfunctions, different production stages and an extended trial period, should also be considered for future research.

## Author Contribution

Nadine Wallauch was involved in sampling and sample preparation, performed flow cytometric and RT-qPCR analyses, analyzed the data, prepared the figures, and wrote the original draft. Sonja Schmucker contributed to the study design and experimental methods, evaluated the results, supervised the work, and revised the manuscript. Tanja Hofmann and Vera Sommerfeld contributed to the revision of the manuscript. Martin Hasselmann provided support and resources for the PCR methodology and revised the manuscript. Korinna Huber and Markus Rodehutscord contributed to the study design and manuscript revision. Volker Stefanski contributed to the study design, supervised the work, and revised the manuscript. All authors read, reviewed, and approved the final version of the manuscript.

## Disclosures

The authors declare that they have no known competing financial interests or personal relationships that could have appeared to influence the work reported in this paper.

## Appendix Supplementary materials

Image, application 1

## Footnotes

Supplementary material associated with this article can be found, in the online version, at doi:10.1016/j.psj.2026.107575.
