# Supplementary material for: Impact of dietary myo-inositol supplements on immune cell distribution and cytokine expression in laying hens of two different strains
Source: Poult Sci. 2026 Aug 5;105(11):107575. doi: 10.1016/j.psj.2026.107575 (PMC13572523; doi:10.1016/j.psj.2026.107575)
Supplement: Supplementary file 1 [file mmc1.pdf]

Supplemental Material

Table S1 Ingredients, calculated and analyzed compositions of experimental diets (already published in Sommerfeld et al., 2025)

Ingredients [g/kg]	**MI0**	**MI1**	**MI2**	**MI3**
Corn	591	590	589	588
Extracted soybean meal	260	260	260	260
Alfalfa meal	30	30	30	30
Soybean oil	20	20	20	20
DL-Methionine	3.5	3.5	3.5	3.5
Monocalcium phosphate	4.7	4.7	4.7	4.7
Limestone, fine	23	23	23	23
Limestone, coarse	54.4	54.4	54.4	54.4
Sodium chloride	3	3	3	3
Choline chloride	1	1	1	1
Sodium bicarbonate	1.9	1.9	1.9	1.9
Vitamin mix[1]	2	2	2	2
Mineral mix[2]	0.5	0.5	0.5	0.5
Myo-inositol[3]	0	1	2	3
TiO_2	5	5	5	5
Calculated [g/kg]				
Myo-inositol	0.4	1.4	2.4	3.4
Phosphorus	4.0	4.0	4.0	4.0
Non phytate-phosphorus	2.2	2.2	2.2	2.2
Calcium	35.0	35.0	35.0	35.0
Crude protein	168	168	168	168
Metabolizable energy [MJ/kg]	11.6	11.6	11.6	11.6
Analyzed [g/kg dry matter]				
Myo-inositol	0.37	1.51	2.76	3.72
Phosphorus	4.4.	4.5	4.4	4.5
Calcium	34.6	36.3	33.3	33.3

[1]Vitamin premix (Miavit GmbH, Essen, Germany), provided per kg of the complete diet: 10,000 IU vitamin A, 3,000 IU vitamin D3, 30 mg vitamin E, 2.4 mg vitamin K3, 100 mcg biotin, 1 mg folic acid, 3 mg vitamin B1, 6 mg vitamin B2, 6 mg vitamin B6, 30 mcg vitamin B12, 50 mg nicotinamide, 14 mg calcium-D-pantothenate.

[2]Trace element premix (Gelamin Gesellschaft für Tierernährung mbH, Memmingen, Germany), provided per kg of complete diet: 80 mg manganese from manganese-(II)oxide, 60 mg zinc from zinc sulfate monohydrate, 25 mg iron from ferrous-(II)-sulfate monohydrate, 7.5 mg copper from cupric-(II)-sulfate pentahydrate, 0.6 mg iodine from calcium iodate, 0.2 mg selenium from sodium selenite.

[3]Thermo Fisher GmbH, Kandel, Germany.

Abbreviations: MI0 = 0 g *myo*-inositol/ kg feed; MI1 = 1 g *myo*-inositol/ kg feed; MI2 = 2 g *myo*-inositol/ kg feed; MI3 = 3 g *myo*-inositol/ kg feed.

Supplemental Material

Table S2 RNA-concentrations extracted from spleen, measured using NanoDrop2000 Spectrophotometer

Sample No.	Inserted material [g]	Nucleid Acid Concentration. [ng/µl]	260/280 ratio	260/230 ratio	Sample Type	Sample No.	Inserted material [g]	Nucleid Acid Concentration [ng/µl]	260/280 ratio	260/230 ratio	Sample Type
Spleen 1	0.0975	2858.3	2.01	1.39	RNA	Spleen 21	0.1030	2900.4	2.05	1.52	RNA
Spleen 2	0.1075	3161.6	2.04	1.66	RNA	Spleen 22	0.0997	2817.8	2.05	1.61	RNA
Spleen 3	0.1074	2453.8	2.04	1.72	RNA	Spleen 23	0.1007	2597.1	2.05	1.59	RNA
Spleen 4	0.1087	2184.3	2.04	1.64	RNA	Spleen 24	0.0988	2377.7	2.05	1.54	RNA
Spleen 5	0.1010	2664.3	2.03	1.68	RNA	Spleen 25	0.0985	2814.4	2.06	1.62	RNA
Spleen 6	0.0935	2455.4	2.03	1.65	RNA	Spleen 26	0.1038	2528.9	2.06	1.67	RNA
Spleen 7	0.0955	2503.8	2.04	1.47	RNA	Spleen 27	0.1049	2896.9	2.07	1.63	RNA
Spleen 8	0.0961	2607.5	2.04	1.54	RNA	Spleen 28	0.0967	2262.3	2.07	1.47	RNA
Spleen 9	0.0960	2427.5	2.03	1.62	RNA	Spleen 29	0.1060	2454.9	2.06	1.64	RNA
Spleen 10	0.1023	2710.5	2.03	1.70	RNA	Spleen 30	0.1062	3414.8	2.04	1.76	RNA
Spleen 11	0.0969	2254.0	2.04	1.55	RNA	Spleen 31	0.0928	2488.5	2.05	1.65	RNA
Spleen 12	0.1088	2696.2	2.04	1.59	RNA	Spleen 32	0.0977	2387.6	2.05	1.72	RNA
Spleen 13	0.1019	2699.4	2.02	1.50	RNA	Spleen 33	0.1035	3109.7	2.04	1.62	RNA
Spleen 14	0.0992	2358.3	2.03	1.82	RNA	Spleen 34	0.0963	2373.6	2.05	1.68	RNA
Spleen 15	0.0974	2677.0	2.03	1.79	RNA	Spleen 35	0.1025	2580.3	2.06	1.70	RNA
Spleen 16	0.0983	2091.1	2.03	1.56	RNA	Spleen 36	0.1016	2379.1	2.04	1.54	RNA
Spleen 17	0.1078	2843.2	2.06	1.58	RNA	Spleen 37	0.0982	2198.4	2.06	1.64	RNA
Spleen 18	0.1018	3288.0	2.06	1.56	RNA	Spleen 388	0.0957	2111.5	2.03	1.52	RNA
Spleen 19	0.1072	3060.7	2.05	1.76	RNA	Spleen 39	0.1055	2651.6	2.04	1.74	RNA
Spleen 20	0.0999	2733.3	2.05	1.67	RNA	Spleen 40	0.1060	2947.5	2.05	1.70	RNA

continues

Table S2 continued

Sample No.	Inserted material [g]	Nucleid Acid Concentration. [ng/µl]	260/280 ratio	260/230 ratio	Sample Type	Sample No.	Inserted material [g]	Nucleid Acid Concentration [ng/µl]	260/280 ratio	260/230 ratio	Sample Type
Spleen 41	0.1011	2532.2	2.06	1.55	RNA	Spleen 61	0.0983	2242.0	2.05	1.50	RNA
Spleen 42	0.1050	2627.7	2.05	1.51	RNA	Spleen 62	0.0973	2238.8	2.06	1.68	RNA
Spleen 43	0.1058	2669.8	2.05	1.68	RNA	Spleen 63	0.0978	2425.1	2.06	1.71	RNA
Spleen 44	0.1051	2799.8	2.06	1.67	RNA	Spleen 64	0.1065	2488.7	2.05	1.58	RNA
Spleen 45	0.1001	2356.5	2.06	1.61	RNA	Spleen 65	0.1000	2576.8	2.05	1.54	RNA
Spleen 46	0.0971	2855.2	2.04	1.47	RNA	Spleen 66	0.0937	2352.8	2.04	1.58	RNA
Spleen 47	0.0958	2480.7	2.05	1.55	RNA	Spleen 67	0.1020	2819.6	2.05	1.65	RNA
Spleen 48	0.1033	3006.3	2.04	1.49	RNA	Spleen 68	0.0988	2062.2	2.04	1.54	RNA
Spleen 49	0.1010	2700.2	2.06	1.68	RNA	Spleen 69	0.1008	2737.8	2.06	1.63	RNA
Spleen 50	0.0937	2568.6	2.05	1.49	RNA	Spleen 70	0.0988	2531.1	2.04	1.55	RNA
Spleen 51	0.1079	2885.1	2.05	1.56	RNA	Spleen 71	0.0958	2589.8	2.05	1.61	RNA
Spleen 52	0.1024	2832.4	2.05	1.63	RNA	Spleen 72	0.0970	2164.3	2.03	1.54	RNA
Spleen 53	0.1067	3231.8	2.04	1.62	RNA	Spleen 73	0.1009	2453.8	2.03	1.47	RNA
Spleen 54	0.0975	2819.1	2.04	1.50	RNA	Spleen 74	0.1067	2644.8	2.05	1.70	RNA
Spleen 55	0.1011	2669.3	2.03	1.41	RNA	Spleen 75	0.1048	2331.2	2.03	1.50	RNA
Spleen 56	0.0943	2376.5	2.06	1.64	RNA	Spleen 76	0.0996	2698.1	2.03	1.53	RNA
Spleen 57	0.1030	3255.2	2.04	1.75	RNA	Spleen 77	0.0979	2433.2	2.03	1.49	RNA
Spleen 58	0.0968	2640.6	2.05	1.61	RNA	Spleen 78	0.0946	2737.2	2.05	1.64	RNA
Spleen 59	0.1065	2725.3	2.05	1.65	RNA	Spleen 79	0.1016	2731.8	2.05	1.66	RNA
Spleen 60	0.0931	2429.2	2.06	1.66	RNA	Spleen 80	0.1006	2726.8	2.04	1.40	RNA

Table S3 RNA-concentrations extracted from cecal tonsils, measured using NanoDrop2000 Spectrophotometer

Sample No.	Inserted material [g]	Nucleid Acid Concentration. [ng/µl]	260/280 ratio	260/230 ratio	Sample Type	Sample No.	Inserted material [g]	Nucleid Acid Concentration [ng/µl]	260/280 ratio	260/230 ratio	Sample Type
CT 1	0.1094	2915.2	2.04	1.58	RNA	CT 21	0.1046	2408.5	2.04	1.50	RNA
CT 2	0.1008	3174	2.05	1.81	RNA	CT 22	0.0950	2668.9	2.04	1.43	RNA
CT 3	0.1007	2572.4	2.03	1.41	RNA	CT 23	0.1088	2041.4	2.02	1.48	RNA
CT 4	0.0706	1554.1	2.03	1.18	RNA	CT 24	0.0948	3946.4	2.02	1.69	RNA
CT 5	0.1080	2730.7	2.03	1.40	RNA	CT 25	0.1092	3380.6	2.04	1.61	RNA
CT 6	0.1035	2790.4	2.04	1.41	RNA	CT 26	0.0981	2529.3	2.04	1.43	RNA
CT 7	0.0882	2035.8	2.05	1.53	RNA	CT 27	0.0964	2000.5	2.03	1.40	RNA
CT 8	0.1062	3316.9	2.04	1.45	RNA	CT 28	0.1024	3344	2.04	1.55	RNA
CT 9	0.0502	1391.8	2.03	1.24	RNA	CT 29	0.1004	3688.7	2.02	1.57	RNA
CT 10	0.0943	3192.8	2.04	1.55	RNA	CT 30	0.1014	3548.8	2.02	1.46	RNA
CT 11	0.0968	2578.5	2.02	1.24	RNA	CT 31	0.0988	2345.7	2.00	1.27	RNA
CT 12	0.0998	2613.3	2.04	1.45	RNA	CT 32	0.1039	2230.1	2.03	1.52	RNA
CT 13	-	-	-	-	-	CT 33	0.0780	1807.7	2.03	1.36	RNA
CT 14	0.1080	2019.5	2.04	1.46	RNA	CT 34	0.1000	2397.3	2.03	1.40	RNA
CT 15	0.0963	2120.7	2.02	1.24	RNA	CT 35	0.0925	1795.8	2.01	1.17	RNA
CT 16	0.1047	2041.2	2.05	1.51	RNA	CT 36	0.1026	1920.7	2.04	1.37	RNA
CT 17	0.0958	2995.7	2.04	1.52	RNA	CT 37	0.1039	2178.8	2.02	1.26	RNA
CT 18	0.1024	2201.7	2.02	1.50	RNA	CT 38	0.0853	1712.2	2.01	1.12	RNA
CT 19	0.1075	3099.2	2.02	1.31	RNA	CT 39	0.1009	2501.2	2.03	1.32	RNA
CT 20	0.0553	2455.2	2.04	1.59	RNA	CT 40	0.1094	2806.1	2.04	1.58	RNA

continues

Table S3 continued

Sample No.	Inserted material [g]	Nucleid Acid Concentration. [ng/µl]	260/280 ratio	260/230 ratio	Sample Type	Sample No.	Inserted material [g]	Nucleid Acid Concentration [ng/µl]	260/280 ratio	260/230 ratio	Sample Type
CT 41	0.0987	2758.7	2.02	1.52	RNA	CT 61	0.0945	2237.2	2.04	1.50	RNA
CT 42	0.0970	2179.7	2.01	1.39	RNA	CT 62	0.1055	3276.5	2.01	1.50	RNA
CT 43	0.0993	3078.8	2.02	1.33	RNA	CT 63	0.0982	3290.1	2.03	1.60	RNA
CT 44	0.0855	2014.3	1.99	1.30	RNA	CT 64	0.0950	2213.1	2.02	1.33	RNA
CT 45	0.0936	2216.5	2.02	1.41	RNA	CT 65	0.1017	1630.3	2.05	1.34	RNA
CT 46	0.1037	2569.8	2.01	1.61	RNA	CT 66	0.1059	2052.7	2.03	1.29	RNA
CT 47	0.0952	2362.3	2.03	1.40	RNA	CT 67	0.0997	3152.6	2.03	1.47	RNA
CT 48	0.0940	2689.6	2.03	1.53	RNA	CT 68	0.1067	2529.2	2.03	1.48	RNA
CT 49	0.1089	3644.3	2.02	1.45	RNA	CT 69	0.1033	2585.6	2.03	1.53	RNA
CT 50	0.1091	3131	2.02	1.46	RNA	CT 70	0.0935	2344	1.99	1.16	RNA
CT 51	0.1011	3669.8	2.03	1.70	RNA	CT 71	0.1012	3344.8	2.01	1.38	RNA
CT 52	0.0951	2342.7	2.02	1.35	RNA	CT 72	0.0772	1927.2	2.01	1.04	RNA
CT 53	0.1046	2560.1	2.02	1.39	RNA	CT 73	0.0972	2127.3	2.01	1.15	RNA
CT 54	0.1086	2417.6	2.03	1.40	RNA	CT 74	0.1003	2795.9	2.02	1.41	RNA
CT 55	0.1036	2521.4	2.01	1.27	RNA	CT 75	0.1033	1903.7	2.04	1.33	RNA
CT 56	0.1004	2866.7	2.03	1.55	RNA	CT 76	-	-	-	-	-
CT 57	0.1040	2359.1	2.03	1.37	RNA	CT 77	0.0983	2328.4	2.04	1.55	RNA
CT 58	0.0970	3078.3	2.04	1.65	RNA	CT 78	0.0718	2010.6	2.02	1.39	RNA
CT 59	0.1086	2147.9	2.01	1.43	RNA	CT 79	0.0914	2604.9	2.03	1.45	RNA
CT 60	0.0947	1959.9	2.02	1.46	RNA	CT 80	0.1083	2784.7	2.02	1.40	RNA

Supplemental Material

Table S4 Integrity of extracted RNA of a representative sample subset, measured on Qubit4 using Qubit RNA IQ Assay Kit

Sample No.	Nucleid Acid concentration. [ng/µl]	260/280 ratio	260/230 ratio	Sample Type	Qubit 4 measurement	Ratio small RNA / large, structured RNA
Spleen 15	2677.0	2.03	1.79	RNA	8.2	18 % / 82 %
Spleen 28	2262.3	2.07	1.47	RNA	8.6	14 % / 86 %
Spleen 33	3109.7	2.04	1.62	RNA	8.2	18 % / 82 %
Spleen 48	3006.3	2.04	1.49	RNA	8.2	18 % / 82 %
Spleen 52	2832.4	2.05	1.63	RNA	7.9	21 % / 79 %
Spleen 56	2376.5	2.06	1.64	RNA	8.0	20 % / 80 %
Spleen 67	2819.6	2.05	1.65	RNA	8.6	14 % / 86 %
Spleen 75	2331.2	2.03	1.50	RNA	8.5	15 % / 85 %
CT 1	2915.2	2.04	1.58	RNA	7.6	24 % / 76 %
CT 33	1807.7	2.03	1.36	RNA	7.8	22 % / 78 %
CT 37	2178.8	2.02	1.26	RNA	7.8	22 % / 78 %
CT 53	2560.1	2.02	1.39	RNA	7.4	26 % / 74 %
CT 56	2866.7	2.03	1.55	RNA	7.7	23 % / 77 %
CT 60	1959.9	2.02	1.46	RNA	7.9	21 % / 79 %
CT 68	2529.2	2.03	1.48	RNA	7.8	22 % / 78 %
CT 78	2010.6	2.02	1.39	RNA	8.0	20 % / 80 %

Supplemental Material

Table S5 Primer sequences for quantitative reverse transcription-PCR

Gene		Primer Sequence	Product size (bp)	NCBI Accession Number
GAPDH	F	GAAGGCTGGGGCTCATCTG	150	NM_204305.1
	R	CAGTTGGTGGTGCACGATG		
ACTB	F	GCTGTGCTGTCCCTGTATGC	210	NM_205518
	R	TTCTCTCTCGGCTGTGGTGG		
PPIA	F	TGACTTTACGCGCCACAACG	165	NM_001166326
	R	TCGGTCTTGGCAGTGCAGAT		
IL-1β	F	TCCTGGAGGAGGTTTTTGAG	93	NM_204524.2
	R	AGGACTGTGAGCGGGTGTAG		
IL-2	F	CCGTGGCTAACTAATCTGCTG	123	AF000631.1
	R	AACGTACATTTTGAGCCCGTA		
IL-4	F	GGAGAGCATCCGGATAGTGAA	194	AJ621249.1
	R	TCAGGAGCTGACGCATGTTG		
IL-6	F	TCTGTTCGCCTTTCAGACCTA	142	HM367074.1
	R	GACCACCTCATCGGGATTTAT		
IL-10	F	CGGGAGCTGAGGGTGAAGT	119	NM_001004414.4
	R	CAGCCAAAGGTCCCCTTAAAC		
IL-12	F	TGAAGGAGTTCCCAGATGC	152	AY262752
	R	CGTCTTGCTTGGCTCTTTATAG		
IL-13	F	ATGACACCAGAGTGGCACAAG	187	AJ621250.1
	R	TAGATCTCATTGTCGGTGCTCG		
IL-17	F	GAAGGTGATACGGCCAGGAC	260	NM_204460.2
	R	AGTTCACGCACCTGGAATGG		
TNF-α	F	CGCTCAGAACGACGTCAA	116	MF000729
	R	GTCGTCCACACCAACGAG		
IFN-γ	F	CTGAAGAACTGGACAGAGAG	264	NM_205149.1
	R	CACCAGCTTCTGTAAGATGC		
SOD1	F	TGGAGACAACACAAATGGGTGTA	94	NM_205064.1
	R	CACGTGCCTATCTGCATCTTTT		
SOD2	F	CCCACATCAGTGCAGAGATCA	124	NM_204211
	R	TGAGCTGTAACATCACCTTTTGC		
iNos	F	TGACTTGGAAAAGAAAGGGATCAA	169	NM_204961.2
	R	GACTTTAGGCTGCCCAGGTTG		
foxp3	F	AGTACGCCACAACCTGAGCCT	134	MT133687
	R	ACTTCAGGTCGCAGTCCCTG		

Table S6 Thermal cycling conditions used on the final qPCR runs according to the manufacturers protocol (Standard Bio Tools)

Step	Flex Six		96.96 IFC	
	Temperature	Time	Temperature	Time
Thermal mixing	25 °C	30 min	70 °C	40 min
	70 °C	60 min	60 °C	30 sec
Hot start	95 °C	1 min	95 °C	1 min
PCR, 30 cycles	96 °C	5 sec	96 °C	5 sec
	60 °C	20 sec	60 °C	20 sec
Melting curve	60 °C	3 sec	60 °C	3 sec
	60-95 °C	1 °C/3 sec	60-95 °C	1 °C/3 sec

[1]Flex Six™ Gene Expression Integrated Fluidic Circuits **(**Standard Bio Tools).
[2]96.96 Integrated Fluidic Circuits (**IFC**), 96.96 Dynamic Array™ IFC for Gene Expression **(**Standard Bio Tools

Supplemental Material

Table S7 Impact of different dietary MI levels and strain on numbers of immune cells per µl blood (×10^3) and heterophil-to-lymphocyte (H:L) ratio

Strain[1]	Diet[2]	Leukocytes	Thrombocytes	Monocytes	Heterophils	Total T Cells	Cytotoxic T Cells	$CD4^+$ T Cells	γδ T Cells	B Cells	H:L Ratio
LB	MI0	91.6 ± 3.7	66.0 ± 3.1	7.12 ± 1.0	24.0 ± 2.7	37.0 ± 1.9	7.52 ± 0.6	19.9 ± 1.1	9.18 ± 0.9	8.47 ± 1.0	0.53 ± 0.06
LSL	MI0	70.7 ± 3.7	48.0 ± 3.1	6.37 ± 0.9	10.4 ±1.2	32.6 ± 1.9	6.68 ± 0.6	14.5 ± 1.1	10.9 ± 1.0	5.61 ± 1.0	0.28 ± 0.03
LB	MI1	93.9 ± 3.7	67.9 ± 3.1	6.54 ± 0.9	27.9 ± 3.1	37.1 ± 1.9	7.11 ± 0.6	20.7 ± 1.1	9.07 ± 0.9	7.03 ± 1.0	0.64 ± 0.08
LSL	MI1	67.2 ± 3.9	44.7 ± 3.1	3.61 ± 0.5	8.55 ± 1.0	33.3 ± 2.1	7.55 ± 0.6	15.4 ± 1.2	9.34 ± 0.9	6.87 ± 1.1	0.22 ± 0.03
LB	MI2	96.8 ± 3.7	69.7 ± 3.1	7.99 ± 1.1	31.0 ± 3.5	35.3 ± 1.9	6.88 ± 0.6	18.9 ± 1.1	9.26 ± 0.9	5.93 ± 1.0	0.76 ± 0.09
LSL	MI2	74.3 ± 3.7	51.0 ± 3.1	5.25 ± 0.7	12.1 ± 1.4	34.2 ± 1.9	7.63 ± 0.6	15.9 ± 1.1	10.3 ± 1.0	9.25 ± 1.0	0.28 ± 0.03
LB	MI3	96.3 ± 3.7	70.8 ± 3.1	8.95 ± 1.3	26.9 ± 3.0	37.2 ± 1.9	7.21 ± 0.6	19.2 ± 1.1	10.5 ± 1.0	7.84 ± 1.0	0.60 ± 0.07
LSL	MI3	76.3 ± 3.7	54.4 ± 3.1	5.08 ± 0.7	10.6 ± 1.2	37.0 ± 1.9	7.74 ± 0.6	16.2 ± 1.1	12.6 ± 1.2	7.85 ± 1.0	0.24 ± 0.03
LB		94.6 ± 1.9[a]	68.6 ± 1.6[a]	7.60 ± 0.7[a]	27.3 ± 1.6[a]	36.6 ± 1.3	7.18 ± 0.4	19.7 ± 0.7[a]	9.49 ± 0.6	7.32 ± 0.6	0.63 ± 0.05[a]
LSL		72.1 ± 2.0[b]	49.5 ± 1.6[b]	4.97 ± 0.5[b]	10.3 ± 0.6[b]	34.3 ± 1.3	7.40 ± 0.4	15.5 ± 0.7[b]	10.7 ± 0.7	7.40 ± 0.6	0.25 ± 0.02[b]
	MI0	81.2 ± 2.7	57.0 ± 2.2	6.73 ± 0.8[a]	15.8 ± 1.3	34.8 ± 1.4	7.10 ± 0.4	17.2 ± 0.8	10.0 ± 0.7	7.04 ± 0.7	0.38 ± 0.03
	MI1	80.5 ± 2.7	56.3 ± 2.3	4.86 ± 0.5[b]	15.4 ± 1.3	35.2 ± 1.4	7.33 ± 0.4	18.1 ± 0.8	9.21 ± 0.6	7.95 ± 0.7	0.38 ± 0.03
	MI2	85.5 ± 2.7	60.3 ± 2.2	6.48 ± 0.7[a]	19.4 ± 1.6	34.7 ± 1.4	7.25 ± 0.4	17.4 ± 0.8	9.76 ± 0.7	7.59 ± 0.7	0.46 ± 0.04
	MI3	86.3 ± 2.7	62.6 ± 2.2	6.74 ± 0.8[a]	16.9 ± 1.4	37.1 ± 1.4	7.47 ± 0.4	17.7 ± 0.8	11.5 ± 0.8	7.84 ± 0.7	0.38 ± 0.03
p-values[3]											
Diet × Strain		0.812	0.730	0.175	0.467	0.556	0.393	0.421	0.770	0.016	0.257
Strain		< 0.001	< 0.001	< 0.001	< 0.001	0.198	0.683	< 0.001	0.175	0.928	< 0.001
Diet		0.285	0.157	0.024	0.187	0.464	0.904	0.829	0.057	0.736	0.207

Data are presented as LSmeans ± SEM.

[1]LB = Lohmann Brown-Classic, LSL = Lohmann LSL-Classic.

[2]MI0 = without MI supplementation, MI1 = 1g supplemented MI/kg, MI2 = 2g supplemented MI/kg, MI3 = 3g supplemented MI/kg feed.

[3]Results of statistical analysis of interactions (diet × strain) and main effects of diet or strain using a linear mixed model are given. In case of significance ($P < 0.05$), significant differences of the post hoc test (Fisher LSD; $P < 0.05$) of interactions (diet × strain) or of the diet as a main effect are presented in detail in Figure 1 of the manuscript. Significant differences of the post hoc test (Fisher LSD; $P < 0.05$) of the diet or strain as main effects are indicated by different lowercase letters.

Supplemental Material

Table S8 Impact of different dietary MI levels and strain on immune cell frequencies in blood, presented as proportions of immune cell subsets among total leukocytes [%]

Strain[1]	Diet[2]	Monocytes	Heterophils	Total T Cells	Cytotoxic T Cells	$CD4^+$ T Cells	γδ T Cells	B Cells
LB	MI0	7.79 ± 1.0	26.7 ± 1.8	40.3 ± 1.9	8.18 ± 0.6	21.8 ± 1.3	10.4 ± 1.1	8.07 ± 1.1
LSL	MI0	9.13 ± 1.1	15.7 ± 1.8	46.5 ± 1.9	9.57 ± 0.6	20.7 ± 1.3	16.3 ± 1.1	7.56 ± 1.0
LB	MI1	7.00 ± 0.9	30.4 ± 1.8	39.6 ± 1.9	7.58 ± 0.6	22.1 ± 1.3	9.93 ± 1.1	6.67 ± 0.9
LSL	MI1	5.64 ± 0.7	13.9 ± 1.9	49.5 ± 2.0	11.3 ± 0.6	23.0 ± 1.4	15.3 ± 1.1	9.57 ± 1.4
LB	MI2	8.34 ± 1.0	33.2 ± 1.8	37.2 ± 1.9	7.17 ± 0.6	20.0 ± 1.3	10.0 ± 1.1	5.95 ± 0.8
LSL	MI2	7.12 ± 0.9	17.3 ± 1.8	46.2 ± 1.9	10.3 ± 0.6	21.6 ± 1.3	14.3 ± 1.1	12.1 ± 1.6
LB	MI3	9.35 ± 1.2	28.4 ± 1.8	38.8 ± 1.9	7.50 ± 0.6	20.1 ± 1.3	11.2 ± 1.1	7.55 ± 1.0
LSL	MI3	6.71 ± 0.8	14.9 ± 1.8	48.6 ± 1.9	10.1 ± 0.6	21.4 ± 1.3	17.1 ± 1.1	9.25 ± 1.2
LB		8.08 ± 0.7	29.7 ± 1.1[a]	39.0 ± 1.1[b]	7.61 ± 0.4[b]	21.0 ± 0.7	10.4 ± 0.8[b]	7.01 ± 0.6[b]
LSL		7.04 ± 0.6	15.4 ± 1.1[b]	47.7 ± 1.1[a]	10.3 ± 0.4[a]	21.7 ± 0.7	15.7 ± 0.8[a]	9.48 ± 0.8[a]
	MI0	8.44 ± 0.9[a]	21.2 ± 1.3	43.4 ± 1.3	8.88 ± 0.4	21.2 ± 0.9	13.3 ± 0.8	7.81 ± 0.7
	MI1	6.28 ± 0.7[b]	22.1 ± 1.4	44.6 ± 1.4	9.42 ± 0.4	22.5 ± 1.0	12.6 ± 0.8	7.99 ± 0.8
	MI2	7.70 ± 0.8[a]	25.2 ± 1.3	41.7 ± 1.3	8.73 ± 0.4	20.8 ± 0.9	12.2 ± 0.8	8.47 ± 0.8
	MI3	7.92 ± 0.8[a]	21.6 ± 1.3	43.7 ± 1.3	8.80 ± 0.4	20.8 ± 0.9	14.1 ± 0.8	8.36 ± 0.8
p-values[3]								
Diet × Strain		0.089	0.347	0.677	0.187	0.691	0.772	0.028
Strain		0.091	< 0.001	< 0.001	< 0.001	0.473	< 0.001	0.015
Diet		0.029	0.072	0.435	0.573	0.453	0.110	0.911

Data are presented as LSmeans ± SEM.

[1]LB = Lohmann Brown-Classic, LSL = Lohmann LSL-Classic.

[2]MI0 = without MI supplementation, MI1 = 1g supplemented MI/kg, MI2 = 2g supplemented MI/kg, MI3 = 3g supplemented MI/kg feed.

[3]Results of statistical analysis of interactions (diet × strain) and main effects of diet or strain using a linear mixed model are given. In case of significance ($P < 0.05$), significant differences of the post hoc test (Fisher LSD; $P < 0.05$) of interactions (diet × strain) or of the diet as a main effect are presented in detail in Figure 1 of the manuscript. Significant differences of the post hoc test (Fisher LSD; $P < 0.05$) of the diet or strain as main effects are indicated by different lowercase letters.

Supplemental Material

Table S9 Impact of different dietary MI levels and strain on numbers of immune cells ($\times 10^6$) per g spleen

Strain[1]	Diet[2]	Leukocytes	Macrophages	NK Cells	Total T Cells	Cytotoxic T Cells	$CD4^+$ T Cells	γδ T Cells	B Cells	$CD4^+$ $CD25^-$ T Cells	$CD4^+$ $CD25^{dim}$ T Cells	$CD4^+$ $CD25^{high}$ T Cells
LB	MI0	1515 ± 70	15.5 ± 1.8	12.9 ± 1.4	907 ± 48	388 ± 25	243 ± 14	218 ± 19	584 ± 42	164 ± 12	41.8 ± 3.3	36.9 ± 3.0
LSL	MI0	1587 ± 70	24.5 ± 1.8	11.5 ± 1.3	1086 ± 48	478 ± 25	233 ± 14	334 ± 19	460 ± 42	147 ± 11	49.6 ± 3.3	35.7 ± 3.0
LB	MI1	1306 ± 70	13.9 ± 1.8	11.8 ± 1.3	844 ± 48	352 ± 25	236 ± 14	201 ± 19	439 ± 42	158 ± 12	43.7 ± 3.3	34.1 ± 3.0
LSL	MI1	1585 ± 70	20.6 ± 1.8	10.3 ± 1.1	1091 ± 48	470 ± 25	244 ± 15	334 ± 19	456 ± 42	158 ± 12	50.5 ± 3.3	34.8 ± 3.0
LB	MI2	1321 ± 70	16.7 ± 1.8	9.3 ± 1.0	849 ± 48	365 ± 25	226 ± 13	204 ± 19	445 ± 42	148 ± 11	42.5 ± 3.3	34.2 ± 3.0
LSL	MI2	1784 ± 70	23.8 ± 1.8	10.8 ± 1.2	1185 ± 48	505 ± 25	256 ± 15	375 ± 19	559 ± 42	160 ± 12	55.2 ± 3.3	41.3 ± 3.0
LB	MI3	1449 ± 70	14.8 ± 1.8	12.4 ± 1.4	902 ± 48	403 ± 25	237 ± 14	211 ± 19	526 ± 42	162 ± 12	39.4 ± 3.3	34.2 ± 3.0
LSL	MI3	1622 ± 70	24.5 ± 1.8	9.86 ± 1.1	1099 ± 48	461 ± 25	229 ± 14	372 ± 19	481 ± 42	141 ± 11	48.7 ± 3.3	40.6 ± 3.0
LB		1398 ± 45[b]	15.2 ± 1.1[b]	11.5 ± 0.7	876 ± 31[b]	377 ± 17[b]	235 ± 10	208 ± 13[b]	498 ± 27	158 ± 8.3	41.8 ± 2.0[b]	34.9 ± 1.7
LSL		1645 ± 45[a]	23.3 ± 1.1[a]	10.6 ± 0.7	1115 ± 31[a]	478 ± 17[a]	240 ± 10	354 ± 13[a]	489 ± 27	151 ± 8.0	51.0 ± 2.0[a]	38.1 ± 1.7
	MI0	1551 ± 49	20.0 ± 1.4	12.2 ± 1.0	996 ± 34	433 ± 18	238 ±10	276 ± 13	522 ± 30	155 ± 8.3	45.7 ± 2.5	36.3 ± 2.1
	MI1	1445 ± 49	17.2 ± 1.4	11.0 ± 0.9	968 ± 34	411 ± 18	240 ±10	268 ± 13	447 ± 30	158 ± 8.4	47.1 ± 2.5	34.5 ± 2.1
	MI2	1552 ± 49	20.2 ± 1.4	10.1 ± 0.8	1017 ± 34	435 ± 18	240 ± 10	290 ± 13	502 ± 30	154 ± 8.2	48.9 ± 2.5	37.8 ± 2.1
	MI3	1535 ± 49	19.6 ± 1.4	11.1 ± 0.9	1001 ± 34	432 ± 18	233 ± 10	291 ± 13	503 ± 30	151 ± 8.0	44.0 ± 2.5	37.4 ± 2.1
p-values[3]												
Diet × Strain		0.018	0.770	0.316	0.240	0.291	0.291	0.300	0.019	0.285	0.778	0.369
Strain		0.001	< 0.001	0.347	< 0.001	< 0.001	0.746	< 0.001	0.798	0.560	< 0.001	0.209
Diet		0.262	0.272	0.343	0.697	0.683	0.907	0.402	0.228	0.905	0.450	0.643

Data are presented as LSmeans ± SEM.

[1]LB = Lohmann Brown-Classic, LSL = Lohmann LSL-Classic.

[2]MI0 = without MI supplementation, MI1 = 1g supplemented MI/kg, MI2 = 2g supplemented MI/kg, MI3 = 3g supplemented MI/kg feed.

[3]Results of statistical analysis of interactions (diet × strain) and main effects of diet or strain using a linear mixed model are given. In case of significance ($P < 0.05$), significant differences of the post hoc test (Fisher LSD; $P < 0.05$) of interactions (diet × strain) or of the diet as a main effect, are presented in detail in Figure 2A of the manuscript. Significant differences of the post hoc test (Fisher LSD; $P < 0.05$) of the diet or strain as main effects are indicated by different lowercase letters.

Supplemental Material

Table S10 Impact of different dietary MI levels and strain on immune cell frequencies in the spleen, presented as proportion of immune cell subsets among total leukocytes [%]

Strain[1]	Diet[2]	Macrophages	NK Cells	Total T Cells	Cytotoxic T Cells	$CD4^+$ T Cells	γδ T Cells	B Cells	$CD4^+$ $CD25^-$ T Cells	$CD4^+$ $CD25^{dim}$ T Cells	$CD4^+$ $CD25^{high}$ T Cells
LB	MI0	0.97 ± 0.09	0.86 ± 0.08	60.9 ± 1.9	26.2 ± 1.2	16.2 ± 0.8	14.7 ± 1.0	37.4 ± 1.9	11.0 ± 0.7	2.73 ± 0.2	2.47 ± 0.2
LSL	MI0	1.52 ± 0.13	0.73 ± 0.07	68.4 ± 1.9	30.1 ± 1.2	14.7 ± 0.7	21.0 ± 1.0	28.9 ± 1.9	9.31 ± 0.6	3.09 ± 0.2	2.26 ± 0.2
LB	MI1	1.04 ± 0.09	0.91 ± 0.09	65.0 ± 1.9	27.1 ± 1.2	18.2 ± 0.9	15.6 ± 1.0	33.2 ± 1.9	12.2 ± 0.8	3.22 ± 0.2	2.67 ± 0.2
LSL	MI1	1.26 ± 0.11	0.65 ± 0.06	68.8 ± 1.9	29.5 ± 1.2	15.5 ± 0.8	21.2 ± 1.0	28.8 ± 1.9	10.0 ± 0.7	3.14 ± 0.2	2.21 ± 0.2
LB	MI2	1.24 ± 0.11	0.72 ± 0.07	64.3 ± 1.9	27.7 ± 1.2	17.4 ± 0.9	15.2 ± 1.0	33.4 ± 1.9	11.4 ± 0.8	3.19 ± 0.2	2.62 ± 0.2
LSL	MI2	1.31 ± 0.12	0.61 ± 0.06	66.3 ± 1.9	28.3 ± 1.2	14.5 ± 0.7	21.1 ± 1.0	31.5 ± 1.9	9.03 ± 0.6	3.07 ± 0.2	2.30 ± 0.2
LB	MI3	0.97 ±0.09	0.86 ± 0.08	62.4 ± 1.9	27.6 ± 1.2	16.5 ± 0.8	14.6 ± 1.0	36.2 ± 1.9	11.3 ± 0.8	2.67 ± 0.2	2.34 ± 0.2
LSL	MI3	1.45 ± 0.13	0.61 ± 0.06	67.9 ± 1.9	28.3 ± 1.2	14.3 ± 0.7	23.0 ± 1.0	29.4 ± 1.9	8.78 ± 0.6	2.98 ± 0.2	2.50 ± 0.2
LB		1.05 ± 0.07[b]	0.84 ± 0.05[a]	63.1 ± 1.1[b]	27.2 ± 0.8	17.1 ± 0.5[a]	15.0 ± 0.7[b]	35.1 ± 1.2[a]	11.5 ± 0.5[a]	2.94 ± 0.1	2.52 ± 0.1
LSL		1.38 ± 0.07[a]	0.65 ± 0.04[b]	67.8 ± 1.1[a]	29.1 ± 0.8	14.7 ± 0.4[b]	21.5 ± 0.7[a]	29.6 ± 1.2[b]	9.27 ± 0.4[b]	3.07 ± 0.1	2.32 ± 0.1
	MI0	1.21 ± 0.08	0.79 ± 0.05	64.6 ± 1.3	28.2 ± 0.8	15.5 ± 0.6	17.8 ± 0.7	33.2 ± 1.3	10.1 ± 0.5	2.91 ± 0.2	2.37 ± 0.1
	MI1	1.14 ± 0.07	0.77 ± 0.05	66.9 ± 1.3	28.3 ± 0.8	16.8 ± 0.6	18.4 ± 0.7	31.0 ± 1.3	11.1 ± 0.5	3.18 ± 0.2	2.44 ± 0.1
	MI2	1.28 ± 0.08	0.66 ± 0.04	65.3 ± 1.3	28.0 ± 0.8	15.9 ± 0.6	18.1 ± 0.7	32.4 ± 1.3	10.1 ± 0.5	3.13 ± 0.2	2.46 ± 0.1
	MI3	1.19 ± 0.07	0.73 ± 0.05	65.1 ± 1.3	28.0 ± 0.8	15.4 ± 0.6	18.8 ± 0.7	32.8 ± 1.3	9.96 ± 0.5	2.82 ± 0.2	2.42 ± 0.1
p-values[3]											
Diet × Strain		0.092	0.555	0.440	0.335	0.802	0.347	0.270	0.897	0.474	0.385
Strain		< 0.001	0.005	0.007	0.108	0.002	< 0.001	0.004	0.002	0.356	0.116
Diet		0.612	0.201	0.583	0.985	0.225	0.703	0.621	0.311	0.228	0.964

Data are presented as LSmeans ± SEM.

[1]LB = Lohmann Brown-Classic, LSL = Lohmann LSL-Classic.

[2]MI0 = without MI supplementation, MI1 = 1g supplemented MI/kg, MI2 = 2g supplemented MI/kg, MI3 = 3g supplemented MI/kg feed.

[3]Results of statistical analysis of interactions (diet × strain) and main effects of diet or strain using a linear mixed model are given. In case of significance ($P < 0.05$), significant differences of the post hoc test (Fisher LSD; $P < 0.05$) of the strain as a main effect are indicated by different lowercase letters.

Supplemental Material

Table S11 Impact of different dietary MI levels and strain on numbers of immune cells ($\times 10^6$) per g cecal tonsil

Strain[1]	Diet[2]	Leukocytes	NK Cells	Total T Cells	Cytotoxic T Cells	$CD4^+$ T Cells	γδ T Cells	B Cells	$CD4^+$ $CD25^-$ T Cells	$CD4^+$ $CD25^{dim}$ T Cells	$CD4^+$ $CD25^{high}$ T Cells
LB	MI0	25.6 ± 5.3	0.53 ± 0.1	17.6 ± 3.5	8.73 ± 1.9	3.94 ± 0.9	3.23 ± 0.8	5.35 ± 1.5	1.78 ± 0.4	1.33 ± 0.3	0.64 ± 0.2
LSL	MI0	35.8 ± 6.2	0.50 ± 0.1	23.9 ± 4.1	10.8 ± 2.1	5.91 ± 1.1	5.47 ± 1.3	9.33 ± 2.6	2.54 ± 0.6	2.23 ± 0.3	0.96 ± 0.2
LB	MI1	23.6 ± 5.1	0.42 ± 0.1	16.0 ± 3.4	8.24 ± 1.8	3.62 ± 0.8	2.24 ± 0.5	5.19 ± 1.4	1.45 ± 0.4	1.22 ± 0.3	0.61 ± 0.2
LSL	MI1	27.8 ± 5.5	0.40 ± 0.1	19.8 ± 3.7	9.42 ± 1.9	4.42 ± 0.9	4.67 ± 1.1	6.34 ± 1.7	1.92 ± 0.5	1.49 ± 0.3	0.83 ± 0.2
LB	MI2	22.3 ± 4.9	0.43 ± 0.1	15.4 ± 3.3	8.16 ± 1.8	3.20 ± 0.8	2.87 ± 0.7	4.95 ± 1.4	1.51 ± 0.4	1.04 ± 0.3	0.45 ± 0.1
LSL	MI2	25.3 ± 5.2	0.36 ± 0.1	17.4 ± 3.5	8.23 ± 1.8	4.16 ± 0.9	3.65 ± 0.9	4.75 ± 1.3	1.58 ± 0.4	1.45 ± 0.3	0.43 ± 0.1
LB	MI3	24.9 ± 5.2	0.62 ± 0.1	17.4 ± 3.5	8.60 ± 1.9	3.39 ± 0.8	3.66 ± 0.9	5.06 ± 1.4	1.28 ± 0.3	1.37 ± 0.3	0.61 ± 0.2
LSL	MI3	31.7 ± 5.9	0.51 ± 0.1	22.1 ± 4.0	9.24 ± 1.9	5.39 ± 1.0	5.95 ± 1.4	7.00 ± 1.9	2.05 ± 0.5	1.94 ± 0.3	0.91 ± 0.2
LB		24.1 ± 3.3	0.49 ± 0.1	16.6 ± 2.2	8.43 ± 1.2	3.53 ± 0.5[b]	2.95 ± 0.5[b]	5.14 ± 0.9	1.49 ± 0.2	1.24 ± 0.2[b]	0.57 ± 0.1
LSL		30.0 ± 3.7	0.44 ± 0.1	20.7 ± 2.4	9.40 ± 1.2	4.94 ± 0.6[a]	4.85 ± 0.8[a]	6.66 ± 1.2	1.99 ± 0.3	1.78 ± 0.2[a]	0.75 ± 0.1
	MI0	30.5 ± 4.5	0.52 ± 0.1	20.6 ± 3.0	9.74 ± 1.5	4.87 ± 0.7	4.20 ± 0.8	7.07 ± 1.5	2.12 ± 0.4	1.78 ± 0.2	0.78 ± 0.1
	MI1	25.7 ± 4.1	0.41 ± 0.1	17.8 ± 2.8	8.82 ± 1.4	4.01 ± 0.7	3.24 ± 0.6	5.74 ± 1.2	1.67 ± 0.3	1.36 ± 0.2	0.71 ± 0.1
	MI2	23.8 ± 4.0	0.39 ± 0.1	16.4 ± 2.6	8.20 ± 1.4	3.67 ± 0.6	3.23 ± 0.6	4.85 ± 1.0	1.54 ± 0.3	1.25 ± 0.2	0.44 ± 0.1
	MI3	28.2 ± 4.3	0.56 ± 0.1	19.7 ± 2.9	8.92 ± 1.4	4.33 ± 0.7	4.66 ± 0.9	5.95 ± 1.3	1.62 ± 0.3	1.66 ± 0.2	0.74 ± 0.1
p-values[3]											
Diet × Strain		0.913	0.978	0.950	0.951	0.870	0.686	0.673	0.825	0.662	0.735
Strain		0.084	0.477	0.074	0.468	0.019	0.004	0.156	0.086	0.006	0.151
Diet		0.522	0.306	0.562	0.844	0.520	0.181	0.502	0.532	0.158	0.055

Data are presented as LSmeans ± SEM.

[1]LB = Lohmann Brown-Classic, LSL = Lohmann LSL-Classic.

[2]MI0 = without MI supplementation, MI1 = 1g supplemented MI/kg, MI2 = 2g supplemented MI/kg, MI3 = 3g supplemented MI/kg feed.

[3]Results of statistical analysis of interactions (diet × strain) and main effects of diet or strain using a linear mixed model are given. In case of significance ($P < 0.05$), significant differences of the post hoc test (Fisher LSD; $P < 0.05$) of the strain as a main effect are indicated by different lowercase letters.

Supplemental Material

Table S12 Impact of different dietary MI levels and strain on immune cell frequencies in the cecal tonsils, presented as proportion of immune cell subsets among total leukocytes [%]

Strain[1]	Diet[2]	NK Cells	Total T Cells	Cytotoxic T Cells	$CD4^+$ T Cells	γδ T Cells	B Cells	$CD4^+$ $CD25^-$ T Cells	$CD4^+$ $CD25^{dim+}$ T Cells	$CD4^+$ $CD25^{high+}$ T Cells
LB	MI0	2.25 ± 0.3	70.0 ± 2.9	33.6 ± 2.3	16.7 ± 1.5	13.6 ± 1.6	26.2 ± 3.0	7.05 ± 0.1	5.05 ± 0.7	2.85 ± 0.3
LSL	MI0	1.45 ± 0.2	67.3 ± 2.9	30.2 ± 2.1	16.9 ± 1.5	16.0 ± 1.9	28.6 ± 3.0	7.08 ± 0.1	5.72 ± 0.8	3.00 ± 0.3
LB	MI1	2.20 ± 0.3	67.0 ± 2.9	32.4 ± 2.2	16.6 ± 1.5	11.9 ± 1.4	28.3 ± 3.0	7.11 ± 0.1	4.64 ± 0.7	3.39 ± 0.3
LSL	MI1	1.51 ± 0.2	71.3 ± 2.9	33.6 ± 2.3	16.2 ± 1.5	17.4 ± 2.0	24.5 ± 3.0	6.76 ± 0.1	5.20 ± 0.7	3.14 ± 0.3
LB	MI2	2.08 ± 0.3	69.5 ± 2.9	35.3 ± 2.4	15.4 ± 1.5	14.0 ± 1.6	25.8 ± 3.0	7.01 ± 0.1	4.44 ± 0.6	2.37 ± 0.3
LSL	MI2	1.79 ± 0.2	71.8 ± 2.9	33.0 ± 2.3	16.5 ± 1.5	18.2 ± 2.1	25.2 ± 3.0	7.23 ± 0.1	4.88 ± 0.7	2.32 ± 0.3
LB	MI3	2.69 ± 0.4	71.6 ± 2.9	35.0 ± 2.4	13.8 ± 1.5	16.0 ± 1.9	23.7 ± 3.0	5.24 ± 0.1	4.40 ± 0.6	2.88 ± 0.3
LSL	MI3	1.69 ± 0.2	70.4 ± 2.9	29.4 ± 2.0	16.8 ± 1.5	19.9 ± 2.3	24.7 ± 3.0	6.12 ± 0.1	5.65 ± 0.8	3.18 ± 0.3
LB		2.30 ± 0.2[a]	69.5 ± 1.7	34.0 ± 1.4	15.6 ± 0.9	13.8 ± 0.9[b]	26.0 ± 1.7	6.49 ± 0.1	4.63 ± 0.4	2.87 ± 0.2
LSL		1.61 ± 0.1[b]	70.2 ± 1.7	31.5 ± 1.3	16.6 ± 0.9	17.8 ± 1.2[a]	25.7 ± 1.7	6.77 ± 0.1	5.35 ± 0.5	2.91 ± 0.2
	MI0	1.81 ± 0.2	68.6 ± 2.1	31.9 ± 1.5	16.8 ± 1.1	14.8 ± 1.3	27.4 ± 2.2	7.07 ± 0.1	5.38 ± 0.5	2.92 ± 0.2[a]
	MI1	1.82 ± 0.2	69.1 ± 2.1	33.0 ± 1.6	16.4 ± 1.1	14.4 ± 1.2	26.4 ± 2.2	6.93 ± 0.1	4.91 ± 0.5	3.27 ± 0.2[a]
	MI2	1.93 ± 0.2	70.7 ± 2.1	34.1 ± 1.7	15.9 ± 1.1	16.0 ± 1.4	25.5 ± 2.2	7.12 ± 0.1	4.65 ± 0.5	2.34 ± 0.2[b]
	MI3	2.14 ± 0.2	71.0 ± 2.1	32.1 ± 1.6	15.3 ± 1.1	17.8 ± 1.5	24.2 ± 2.2	5.64 ± 0.1	4.98 ± 0.5	3.03 ± 0.2[a]
p-values[3]										
Diet × Strain		0.618	0.554	0.399	0.585	0.225	0.709	0.787	0.925	0.735
Strain		0.003	0.781	0.209	0.433	0.008	0.903	0.618	0.281	0.895
Diet		0.565	0.774	0.690	0.706	0.779	0.699	0.126	0.723	0.006

Data are presented as LSmeans ± SEM.

[1]LB = Lohmann Brown-Classic, LSL = Lohmann LSL-Classic.

[2]MI0 = without MI supplementation, MI1 = 1g supplemented MI/kg, MI2 = 2g supplemented MI/kg, MI3 = 3g supplemented MI/kg feed.

[3]Results of statistical analysis of interactions (diet × strain) and main effects of diet or strain using a linear mixed model are given. In case of significance ($P < 0.05$), significant differences of the post hoc test (Fisher LSD; $P < 0.05$) of interactions (diet × strain) or of the diet as a main effect, are presented in detail in Figure 2B of the manuscript. Significant differences of the post hoc test (Fisher LSD; $P < 0.05$) of the diet or strain as main effects are indicated by different lowercase letters.

Table S13 Impact of different dietary MI levels and strain on antibody concentration in plasma and bile and on splenocyte proliferation

Strain[1]	Diet[2]	Plasma IgY [mg/ml]	Plasma IgM [µg/ml]	Plasma IgA [µg/ml]	Bile IgA [mg/ml]	ConA ΔCPM	PWM ΔCPM
LB	MI0	7.76 ± 0.6	535 ± 48	351 ± 26	35.3 ± 5.9	1493 ± 205	492 ± 144
LSL	MI0	7.76 ± 0.6	736 ± 48	248 ± 28	32.1 ± 5.6	1818 ± 249	2013 ± 587
LB	MI1	6.96 ± 0.5	624 ± 48	393 ± 26	37.5 ± 6.3	1889 ± 259	543 ± 158
LSL	MI1	9.48 ± 0.7	669 ± 48	282 ± 26	46.1 ± 7.7	1631 ± 223	1737 ± 507
LB	MI2	7.77 ± 0.6	655 ± 48	385 ± 26	31.4 ± 5.2	1873 ± 257	852 ± 249
LSL	MI2	8.53 ± 0.7	754 ± 48	310 ± 26	34.2 ± 5.7	1900 ± 260	2074 ± 605
LB	MI3	7.49 ± 0.6	614 ± 48	399 ± 26	38.2 ± 6.4	2360 ± 323	919 ± 268
LSL	MI3	8.02 ± 0.6	715 ± 48	242± 26	46.1 ± 7.7	1668 ± 229	1824 ± 532
LB		7.49 ± 0.3[b]	607 ± 28[b]	382 ± 15[a]	35.5 ± 3.0	1879 ± 129	676 ± 102[b]
LSL		8.42 ± 0.3[a]	718 ± 28[a]	270 ± 15[b]	39.1 ± 3.3	1751 ± 120	1907 ± 288[a]
	MI0	7.76 ± 0.4	635 ± 35	299 ± 19	33.6 ± 4.1	1648 ± 160	995 ± 205
	MI1	8.12 ± 0.4	646 ± 35	337 ± 18	41.5 ± 4.9	1755 ± 170	971 ± 200
	MI2	8.14 ± 0.4	704 ± 35	348 ± 18	32.8 ± 3.9	1887 ± 183	1329 ± 274
	MI3	7.75 ± 0.4	665 ± 35	321 ± 18	42.0 ± 5.0	1984 ± 192	1295 ± 267
p-values[3]							
Diet × Strain		0.210	0.385	0.420	0.804	0.242	0.617
Strain		0.033	0.008	< 0.001	0.419	0.468	< 0.001
Diet		0.853	0.449	0.246	0.294	0.550	0.572

Data are presented as LSmeans ± SEM.

[1]LB = Lohmann Brown-Classic, LSL = Lohmann LSL-Classic.

[2]MI0 = without MI supplementation, MI1 = 1g supplemented MI/kg, MI2 = 2g supplemented MI/kg, MI3 = 3g supplemented MI/kg feed.

[3]Results of statistical analysis of interactions (diet × strain) and main effects of diet or strain using a linear mixed model are given. In case of significance (P < 0.05), significant differences of the post hoc test (Fisher LSD; P < 0.05) of the strain as a main effect are indicated by different lowercase letters.

Table S14 Impact of different dietary MI levels and strain on gene expression of pro- and anti-inflammatory cytokines, foxp3 and genes related to oxidative stress reactions in the cecal tonsils

Strain[1]	Diet[2]	IL-1β	IL-2	IL-6	IL-10<br>[$2^{-\Delta Cq} \times 10^{-4}$]	IL-12	IL-13	IL-17
LB	MI0	8.26 ± 0.9	8.16 ± 0.9	0.20 ± 0.06	0.51 ± 0.1	0.40 ± 0.1	1.33 ± 0.3	3.75 ± 1.0
LSL	MI0	9.70 ± 1.0	9.62 ± 1.0	0.20 ± 0.06	1.36 ± 0.3	0.39 ± 0.1	1.09 ± 0.3	2.43 ± 0.7
LB	MI1	9.53 ± 1.0	9.42 ± 1.0	0.15 ± 0.04	0.98 ± 0.2	0.45 ± 0.1	1.43 ± 0.4	3.91 ± 1.0
LSL	MI1	8.10 ± 0.9	8.05 ± 0.8	0.17 ± 0.05	1.45 ± 0.3	0.42 ± 0.1	0.76 ± 0.2	2.27 ± 0.6
LB	MI2	9.22 ± 1.0	9.19 ± 1.0	0.17 ± 0.05	1.04 ± 0.2	0.49 ± 0.1	2.28 ± 0.6	3.30 ± 0.9
LSL	MI2	11.0 ± 1.1	10.9 ± 1.1	0.27 ± 0.08	1.48 ± 0.3	0.43 ± 0.1	0.92 ± 0.2	3.66 ± 0.9
LB	MI3	7.96 ± 0.9	7.92 ± 0.9	0.16 ± 0.05	0.83 ± 0.2	0.56 ± 0.1	1.45 ± 0.4	5.69 ± 1.6
LSL	MI3	10.3 ± 1.1	10.2 ± 1.1	0.17 ± 0.05	1.46 ± 0.3	0.42 ± 0.1	0.58 ± 0.2	3.20 ± 0.8
LB		8.72 ± 0.6	8.65 ± 0.6	0.17 ± 0.03	0.82 ± 0.1[b]	0.47 ± 0.05	1.58 ± 0.2[a]	4.08 ± 0.6
LSL		9.71 ± 0.6	9.64 ± 0.6	0.20 ± 0.03	1.44 ± 0.2[a]	0.42 ± 0.04	0.81 ± 0.1[b]	2.84 ± 0.4
	MI0	8.96 ± 0.7	8.86 ± 0.7	0.20 ± 0.04	0.85 ± 0.1	0.40 ± 0.1	1.20 ± 0.2	3.03 ± 0.6
	MI1	8.79 ± 0.7	8.71 ± 0.7	0.16 ± 0.03	1.19 ± 0.2	0.44 ± 0.1	1.04 ± 0.2	2.99 ± 0.6
	MI2	10.1 ± 0.8	10.0 ± 0.8	0.22 ± 0.05	1.25 ± 0.2	0.46 ± 0.1	1.45 ± 0.3	3.48 ± 0.7
	MI3	9.06 ± 0.7	9.01 ± 0.7	0.17 ± 0.04	1.11 ± 0.2	0.48 ± 0.1	0.91 ± 0.2	4.29 ± 0.8
p-values[3]								
Diet × Strain		0.170	0.173	0.871	0.335	0.878	0.437	0.510
Strain		0.133	0.125	0.382	< 0.001	0.336	0.003	0.100
Diet		0.536	0.508	0.725	0.148	0.672	0.321	0.464

continues

Supplemental Material

Table S14 continued

Strain[1]	Diet[2]	TNF-α	IFN-γ	SOD1	SOD2	iNos	Foxp3	IL-4[4]
				$[2^{-\Delta Cq} \times 10^{-4}]$				[%/100]
LB	MI0	4.41 ± 0.6	3.01 ± 0.4	953 ± 55	303 ± 16	86.4 ± 6.9	28.7 ± 4.6	0.50 ± 0.2
LSL	MI0	6.88 ± 1.0	2.47 ± 0.4	892 ± 51	251 ± 14	89.0 ± 7.1	44.7 ± 7.2	0.20 ± 0.1
LB	MI1	5.80 ± 0.8	4.39 ± 0.6	1019 ± 59	305 ± 16	80.9 ± 6.4	37.7 ± 6.1	0.40 ± 0.2
LSL	MI1	6.02 ± 0.9	2.51 ± 0.4	950 ± 55	273 ± 15	91.1 ± 7.3	32.7 ± 5.3	0.10 ± 0.1
LB	MI2	6.36 ± 1.0	4.61 ± 0.6	894 ± 55	284 ± 16	85.1 ± 7.1	42.4 ± 7.2	0.30 ± 0.2
LSL	MI2	6.82 ± 1.0	2.67 ± 0.4	852 ± 49	252 ± 14	102.2 ± 8.1	40.5 ± 6.5	0.50 ± 0.2
LB	MI3	5.17 ± 0.8	3.65 ± 0.6	1040 ± 63	325 ± 19	75.4 ± 6.6	26.6 ± 4.5	0.20 ± 0.1
LSL	MI3	7.66 ± 1.1	2.70 ± 0.4	837 ± 48	246 ± 13	100.4 ± 8.0	50.9 ± 8.2	0.40 ± 0.2
LB		5.38 ± 0.5[b]	3.87 ± 0.3[a]	975 ± 30[a]	304 ± 9.1[a]	81.8 ± 4.5[b]	33.2 ± 3.5[b]	0.34 ± 0.1
LSL		6.82 ± 0.6[a]	2.59 ± 0.2[b]	882 ± 26[b]	256 ± 7.5[b]	95.5 ± 5.1[a]	41.7 ± 4.3[a]	0.27 ± 0.1
	MI0	5.51 ± 0.6	2.73 ± 0.3	922 ± 38	276 ± 11	87.7 ± 5.5	35.9 ± 4.5	0.33 ± 0.1
	MI1	5.91 ± 0.7	3.33 ± 0.3	984 ± 41	288 ± 11	85.9 ± 5.4	35.1 ± 4.4	0.21 ± 0.1
	MI2	6.58 ± 0.7	3.53 ± 0.4	873 ± 37	268 ± 11	93.2 ± 6.0	41.4 ± 5.3	0.39 ± 0.1
	MI3	6.29 ± 0.7	3.15 ± 0.3	933 ± 40	283 ± 11	87.0 ± 5.7	36.8 ± 4.7	0.29 ± 0.1
p-values[3]								
Diet × Strain		0.264	0.454	0.452	0.397	0.331	0.024	0.129
Strain		0.012	< 0.001	0.017	< 0.001	0.010	0.033	0.523
Diet		0.545	0.291	0.239	0.552	0.656	0.679	0.700

Data are presented as $2^{-\Delta Cq}$-transformed LSmeans ± SEM of ΔCq values, with ΔCq = mean $Cq_{\text{(gen of interest)}}$ – mean $Cq_{\text{(reference genes)}}$.

[1]LB = Lohmann Brown-Classic, LSL = Lohmann LSL-Classic.

[2]MI0 = without MI supplementation, MI1 = 1g supplemented MI/kg, MI2 = 2g supplemented MI/kg, MI3 = 3g supplemented MI/kg feed.

[3]Results of statistical analysis of interactions (diet × strain) and main effects of diet or strain using a linear mixed model or generalised linear model are given. In case of significance (P < 0.05), significant differences of the post hoc test (Fisher LSD; P < 0.05) of interactions (diet × strain) or of the diet as a main effect, are presented in detail in Figure 3 of the manuscript. Significant differences of the post hoc test (Fisher LSD; P < 0.05) of the diet or strain as main effects are indicated by different lowercase letters.

[4]For IL-4, relative proportion [%] of expression frequency is given (n=80).

Table S15 Impact of different dietary MI levels and strain on gene expression of pro- and anti-inflammatory cytokines, foxp3 and genes related to oxidative stress reactions in the spleen

Strain[1]	Diet[2]	IL-1β	IL-2	IL-6	IL-12	TNF-α	IFN-γ	SOD1	SOD2	iNos	Foxp3	IL-10[4]
						$[2^{-\Delta Cq} \times 10^{-4}]$						[%/100]
LB	MI0	2.05 ± 0.3	3.39 ± 0.6	0.37 ± 0.1	1.59 ± 0.4	7.32 ± 0.9	0.91 ± 0.2	804 ± 33	276 ± 8.7	65.6 ± 6.1	123 ± 17	0.18 ± 0.1
LSL	MI0	8.02 ± 1.3	5.01 ± 0.9	0.61 ± 0.1	1.67 ± 0.4	10.2 ± 1.3	0.71 ± 0.2	828 ± 34	260 ± 8.2	85.9 ± 8.0	130 ± 18	0.18 ± 0.1
LB	MI1	3.02 ± 0.5	4.92 ± 0.8	0.44 ± 0.1	2.11 ± 0.5	8.91 ± 1.1	1.32 ± 0.3	781 ± 33	249 ± 7.8	77.0 ± 7.2	173 ± 24	0.28 ± 0.2
LSL	MI1	11.2 ± 1.8	5.32 ± 0.9	0.48 ± 0.1	1.54 ± 0.3	13.4 ± 1.7	0.65 ± 0.2	843 ± 35	259 ± 8.2	105.3 ± 9.8	152 ± 21	0.50 ± 0.2
LB	MI2	2.05 ± 0.3	5.34 ± 0.9	0.56 ± 0.1	1.82 ± 0.4	7.87 ± 1.0	1.12 ± 0.3	799 ± 33	263 ± 8.3	66.7 ± 6.2	159 ± 22	0.18 ± 0.1
LSL	MI2	10.1 ± 1.6	4.73 ± 0.8	0.33 ± 0.1	2.42 ± 0.5	11.8 ± 1.5	1.10 ± 0.3	808 ± 34	252 ± 7.9	94.0 ± 8.8	136 ± 19	0.72 ± 0.2
LB	MI3	2.84 ± 0.5	4.03 ± 0.7	0.58 ± 0.2	2.42 ± 0.4	7.95 ± 1.0	1.48 ± 0.4	773 ± 32	258 ± 8.1	73.9 ± 6.9	147 ± 20	0.18 ± 0.1
LSL	MI3	11.6 ± 1.9	7.06 ± 1.2	0.54 ± 0.1	2.58 ± 0.6	12.5 ± 1.6	0.77 ± 0.2	876 ± 36	258 ± 8.1	115.3 ± 10.8	142 ± 20	0.50 ± 0.2
LB		2.45 ± 0.2[b]	4.35 ± 0.4	0.48 ± 0.1	1.96 ± 0.3	7.99 ± 0.5[b]	1.19 ± 0.2	789 ± 19[b]	261 ± 4.9	70.7 ± 3.6[b]	149 ± 10	0.20 ± 0.1[b]
LSL		10.1 ± 0.9[a]	5.46 ± 0.5	0.48 ± 0.1	2.00 ± 0.3	11.9 ± 0.8[a]	0.79 ± 0.1	838 ± 21[a]	257 ± 4.9	99.5 ± 5.0[a]	140 ± 10	0.46 ± 0.1[a]
	MI0	4.05 ± 0.5	4.13 ± 0.5	0.47 ± 0.1	1.63 ± 0.3	8.66 ± 0.8	0.80 ± 0.1	816 ± 26	268 ± 6.4	75.1 ± 5.0	126 ± 12	0.18 ± 0.1
	MI1	5.81 ± 0.7	5.11 ± 0.6	0.46 ± 0.1	1.81 ± 0.3	10.9 ± 1.0	0.93 ± 0.2	812 ± 25	254 ± 6.1	90.1 ± 6.0	162 ± 16	0.39 ± 0.1
	MI2	4.55 ± 0.5	5.03 ± 0.6	0.43 ± 0.1	2.10 ± 0.3	9.65 ± 0.9	1.11 ± 0.2	804 ± 25	257 ± 6.1	79.2 ± 5.2	147 ± 14	0.43 ± 0.2
	MI3	5.73 ± 0.7	5.33 ± 0.6	0.56 ± 0.1	2.50 ± 0.4	9.95 ± 0.9	1.07 ± 0.2	823 ± 26	258 ± 6.2	92.3 ± 6.1	144 ± 14	0.32 ± 0.1
p-values[3]												
Diet × Strain		0.817	0.173	0.193	0.516	0.974	0.450	0.470	0.350	0.797	0.874	0.466
Strain		< 0.001	0.059	0.994	0.910	< 0.001	0.067	0.032	0.443	< 0.001	0.495	0.027
Diet		0.060	0.449	0.764	0.207	0.312	0.539	0.941	0.278	0.077	0.357	0.426

Data are presented as $2^{-\Delta Cq}$-transformed LSmeans ± SEM of ΔCq values, with $\Delta Cq = \text{mean } Cq_{\text{(gen of interest)}} - \text{mean } Cq_{\text{(reference genes)}}$.

[1]LB = Lohmann Brown-Classic, LSL = Lohmann LSL-Classic.

[2]MI0 = without MI supplementation, MI1 = 1g supplemented MI/kg, MI2 = 2g supplemented MI/kg, MI3 = 3g supplemented MI/kg feed.

[3]Results of statistical analysis of interactions (diet × strain) and main effects of diet or strain using a linear mixed model or generalised linear model are given. In case of significance (P < 0.05), significant differences of the post hoc test (Fisher LSD; P < 0.05) of the strain as a main effect are indicated by different lowercase letters.

[4]For IL-10 relative proportion [%] of expression frequency is given (n=80).

Supplemental Material

Table S16 Impact of different dietary MI levels and strain on gene expression of pro- and anti-inflammatory cytokines, foxp3 and genes related to oxidative stress reactions in the oviduct

Strain[1]	Diet[2]	IL-1β	IL-2	IL-6	IL-12	IL-13	TNF-α	IFN-γ	SOD1	SOD2	iNos	Foxp3	IL-10[4]
							$[2^{-\Delta Cq} \times 10^{-4}]$						[%/100]
LB	MI0	0.31 ± 0.1	2.18 ± 0.3	13.2 ± 2.1	0.95 ± 0.2	0.22 ± 0.06	0.35 ± 0.05	0.24 ± 0.05	1713 ± 77	1070 ± 47	213 ± 11	4.38 ± 06.	0.10 ± 0.1
LSL	MI0	0.42 ± 0.1	1.59 ± 0.2	13.3 ± 2.2	0.44 ± 0.1	0.20 ± 0.04	0.35 ± 0.05	0.26 ± 0.05	1804 ± 85	815 ± 38	214 ± 11	4.79 ± 0.7	0.19 ± 0.1
LB	MI1	0.46 ± 0.1	2.02 ± 0.3	13.5 ± 2.1	2.99 ± 0.6	0.16 ± 0.03	0.33 ± 0.05	0.40 ± 0.07	1908 ± 86	1095 ± 48	225 ± 11	4.21 ± 0.6	0.40 ± 0.2
LSL	MI1	0.41 ± 0.1	2.17 ± 0.3	14.3 ± 2.2	0.45 ± 0.1	0.16 ± 0.03	0.42 ± 0.06	0.33 ± 0.06	1831 ± 82	840 ± 37	223 ± 11	5.17 ± 0.8	0.29 ± 0.2
LB	MI2	0.30 ± 0.1	1.82 ± 0.3	11.1 ± 1.8	2.62 ± 0.5	0.12 ± 0.02	0.39 ± 0.06	0.33 ± 0.06	1926 ± 86	1109 ± 49	233 ± 12	3.94 ± 0.6	0.20 ± 0.1
LSL	MI2	0.36 ± 0.1	1.79 ± 0.2	14.4 ± 2.2	0.46 ± 0.1	0.19 ± 0.04	0.35 ± 0.05	0.34 ± 0.07	1807 ± 81	869 ± 38	236 ± 12	4.09 ± 0.6	0.19 ± 0.1
LB	MI3	0.36 ± 0.1	2.12 ± 0.3	15.9 ± 2.4	2.06 ± 0.4	0.15 ± 0.03	0.48 ± 0.07	0.34 ± 0.06	1734 ± 78	960 ± 42	222 ± 11	4.52 ± 0.7	0.40 ± 0.2
LSL	MI3	0.58 ± 0.1	2.34 ± 0.3	10.1 ± 1.6	0.61 ± 0.1	0.13 ± 0.03	0.34 ± 0.05	0.24 ± 0.05	1871 ± 84	875 ± 39	211 ± 11	4.94 ± 0.7	0.30 ± 0.2
LB		0.35 ± 0.1	2.03 ± 0.1	13.3 ± 1.2	2.00 ± 0.2[a]	0.16 ± 0.02	0.38 ± 0.03	0.32 ± 0.04	1818 ± 50	1057 ± 32[a]	223 ± 6.3	4.26 ± 0.3	0.25 ± 0.1
LSL		0.43 ± 0.1	1.95 ± 0.1	12.9 ± 1.2	0.48 ± 0.1[b]	0.17 ± 0.02	0.36 ± 0.03	0.29 ± 0.04	1828 ± 50	849 ± 26[b]	221 ± 6.3	4.73 ± 0.3	0.24 ± 0.1
	MI0	0.36 ± 0.1	1.86 ± 0.2	13.2 ± 1.6	0.65 ± 0.1[b]	0.21 ± 0.03	0.35 ± 0.04	0.25 ± 0.04	1758 ± 60	934 ± 32	214 ± 7.9	4.58 ± 0.5	0.14 ± 0.1
	MI1	0.43 ± 0.1	2.10 ± 0.2	13.9 ± 1.6	1.22 ± 0.2[a]	0.16 ± 0.02	0.38 ± 0.04	0.36 ± 0.05	1869 ± 63	959 ± 32	224 ± 8.0	4.67 ± 0.5	0.34 ± 0.1
	MI2	0.33 ± 0.1	1.81 ± 0.2	12.7 ± 1.5	1.14 ± 0.2[a]	0.15 ± 0.02	0.37 ± 0.04	0.34 ± 0.05	1866 ± 63	982 ± 33	234 ± 8.4	4.01 ± 0.4	0.19 ± 0.1
	MI3	0.46 ± 0.1	2.23 ± 0.2	12.7 ± 1.5	1.14 ± 0.2[a]	0.14 ± 0.02	0.41 ± 0.04	0.29 ± 0.04	1801 ± 60	916 ± 31	217 ± 7.8	4.72 ± 0.5	0.35 ± 0.1
p-values[3]													
Diet × Strain		0.588	0.373	0.105	0.047	0.438	0.252	0.640	0.259	0.068	0.909	0.947	0.860
Strain		0.186	0.674	0.789	< 0.001	0.616	0.580	0.542	0.859	< 0.001	0.793	0.316	0.961
Diet		0.399	0.337	0.912	0.033	0.292	0.759	0.191	0.395	0.286	0.225	0.660	0.353

Data are presented as $2^{-\Delta Cq}$-transformed LSmeans ± SEM of ΔCq values, with ΔCq = mean Cq $_{\text{(gen of interest)}}$ – mean Cq $_{\text{(reference genes)}}$.

[1]LB = Lohmann Brown-Classic, LSL = Lohmann LSL-Classic.

[2]MI0 = without MI supplementation, MI1 = 1g supplemented MI/kg, MI2 = 2g supplemented MI/kg, MI3 = 3g supplemented MI/kg feed.

[3]Results of statistical analysis of interactions (diet × strain) and main effects of diet or strain using a linear mixed model or generalized linear model are given. In case of significance (P < 0.05), significant differences of the post hoc test (Fisher LSD; P < 0.05) of interactions (diet × strain) or of the diet as a main effect, are presented in detail in Figure 3 of the manuscript. Significant differences of the post hoc test (Fisher LSD; P < 0.05) of the diet or strain as main effects are indicated by different lowercase letters.

[4]For IL-10 relative proportion [%] of expression frequency is given (n=80).
